# Repurposing felodipine via hyaluronic acid-coated cetylosomes (HCCs) for parenteral active targeting of cancer: Box-Behnken statistical optimization, in vitro characterization, and in vivo studies

**DOI:** 10.1007/s13346-026-02090-0

**Published:** 2026-04-06

**Authors:** Rania Moataz El-Dahmy, Moaz A. Eltabeeb, Sahar M. Fayez, Soad Z. El-Emam, Nagwa M. El Adl, Mona M. Saber, Abdurrahman M. Fahmy

**Affiliations:** 1 Department of Pharmaceutics and Industrial Pharmacy, Faculty of Pharmacy, October 6 University, Cairo, Egypt; 2 Department of Industrial Pharmacy, College of Pharmaceutical Sciences and Drug Manufacturing, Misr University for Science and Technology, Giza, Egypt; 3 Department of Pharmacology and Toxicology, Faculty of Pharmacy, October 6 University, Cairo, Egypt; 4 Department of Pharmacology and Toxicology, Faculty of Pharmacy, Cairo University, Cairo, Egypt; 5 Department of Clinical Pharmacy, Faculty of Pharmacy, E-JUST University, Alexandria, Egypt; 6 Department of Pharmaceutics and Industrial Pharmacy, Faculty of Pharmacy, Cairo University, Cairo, Egypt; 7 School of Life and Medical Sciences, University of Hertfordshire Hosted By Global Academic Foundation, New Administrative Capital, Cairo, Egypt

**Keywords:** Felodipine, Hyaluronic Acid-Coated Cetylosomes, Anticancer, Box-Behnken Design, Cell-line, In vivo

## Abstract

Felodipine (FEL), an antihypertensive drug, is being repurposed as an anticancer drug. It has low oral bioavailability due to its extensive metabolism in the liver and poor water solubility. This study aimed to develop hyaluronic acid-coated cetylosomes (HCCs) for parenteral delivery of FEL to boost its solubility and anticancer efficacy. FEL-HCCs were prepared by the thin-film hydration technique based on Box-Behnken design. Amounts of cetyl alcohol, Brij 97, and hyaluronic acid were the independent factors. The optimized HCC (OHCC) formula which showed the highest desirability value (0.626), was composed of 100 mg Brij 97, 32.44 mg CA, and 10 mg HA. It showed the minimum vesicle size (156.72 nm) and polydispersity index (0.134), and the maximum entrapment efficiency% (72.04%) and zeta potential (-30.58 mV). After lyophilization and sterilization of the OHCC formula, it showed a 2.01-fold enhancement in FEL release compared to the market tablet, with no significant difference in release before and after lyophilization or sterilization. The sterilized OHCC was stable for six months. The OHCC showed an enhanced cytotoxicity against MCF-7 cells with 6.61-fold compared to pure FEL powder. Additionally, the OHCC potentiated the antiproliferative effect of doxorubicin, as evidenced by a raised Bax/Bcl-2 ratio and caspase 9 content, and suppressed VEGF levels. In vivo*,* the OHCC markedly reduced tumor growth, particularly in combination with doxorubicin. The histopathological investigation supported the apoptotic and necrotic death of cancer cells. These findings suggest that HCCs represent a promising nanocarrier system for the targeted parenteral delivery of FEL in cancer therapy.

**Graphical abstract:**

**Figure:**
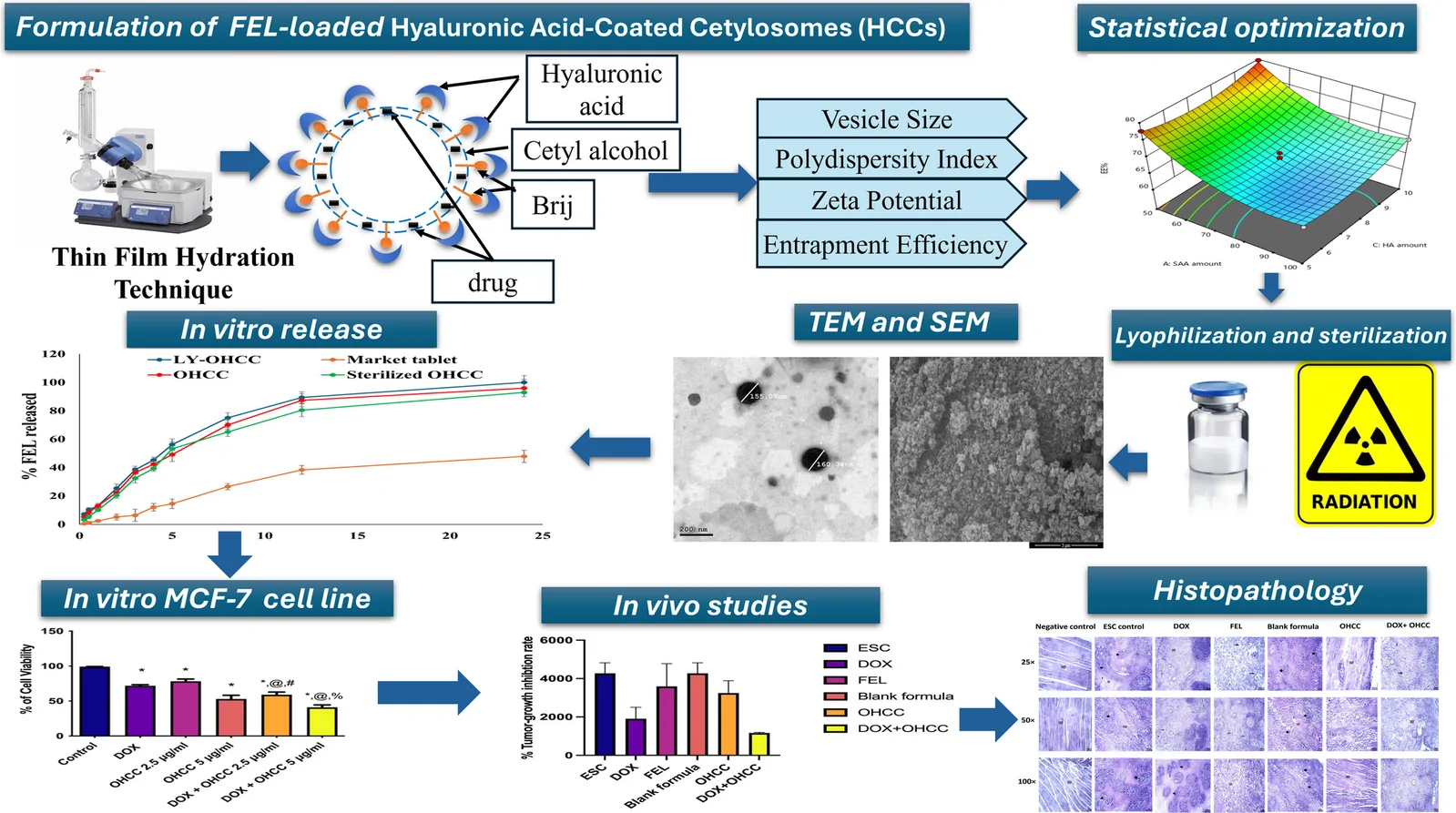

**Supplementary Information:**

The online version contains supplementary material available at https://doi.org/10.1007/s13346-026-02090-0.

## Introduction

Felodipine (FEL), a dihydropyridine calcium-channel blocker (CCB), is commonly used in the treatment of angina pectoris and hypertension [1]. FEL includes 1,4-Dihydropyridines (1,4 DHPs) that are significant members of the calcium channel blockers (CCBs) family [2]. 1,4-DHPs have many therapeutic uses as antihypertensive, antithrombotic, anti-inflammatory, analgesic activities, antiviral, and anticancer [3]. Hence, this study investigates the potential efficacy of FEL as an anticancer drug.

FEL is a Biopharmaceutical Classification System (BCS) class II. It has a very low oral bioavailability (15%) due to its extensive metabolism in the gut and the liver, poor water solubility, and being excreted almost entirely as a metabolite [1]. So, an alternative route of administration was needed to avoid FEL oral problems, like the parenteral route of administration that achieves fast drug delivery to the systemic circulation, achieving rapid therapeutic effect, and avoiding the drug metabolism in the gut and liver, increasing its therapeutic concentration available at the action site, potentiating its bioavailability and anticancer efficacy.

Nanocarriers are promising strategies that can improve the low water solubility of FEL. Several attempts have been made to improve FEL aqueous solubility by preparing it in the form of nanosuspension, solid lipid nanoparticle, microemulsion, and polymeric nanocapsules [1, 4–6]. Moreover, nanocarriers can evade uptake by the reticuloendothelial system, thereby prolonging the circulation of the drug in the bloodstream. Also, nanocarriers with a certain diameter range and surface characteristics are not eliminated easily, accumulating in the tumor region [7].

Cetylosomes (cetyl alcohol-based nanovesicles) are novel nanovesicles that are prepared using cetyl alcohol in combination with nonionic or ionic surfactants that can form stable unilamellar vesicles. Cetyl alcohol (CA) is a fatty base, and it is an amphiphilic molecule associated with a polar hydroxyl head with an alkyl chain tail. It is used to form a bilayer to boost the lipophilic drugs’ entrapment and increase the vesicles’ stability [8].

Elsayed et al. used cetyl alcohol in preparing an in situ Forming nanovesicular system loaded with Rosuvastatin Calcium. They stated that cetyl alcohol enhanced drug entrapment by imparting a lipophilic environment, improved lymphatic uptake, and boosted drug bioavailability by threefold compared to the market tablet [9]. Nasr et al. used Cetyl alcohol to prepare fatty alcohol-based nanovesicles loaded with celecoxib. They stated that cetyl alcohol increased the drug entrapment, system stability, sustainment of drug release, and the optimized formula’s bioavailability by twofold compared to the market product [10]. El-Dahmy et al. used cetyl alcohol in the preparation of lyophilized lacidipine-loaded fatty-based nanovesicles as orally fast disintegrating sponge delivery system. They stated that cetyl alcohol enhanced the drug entrapment and the lyophilized sponge’s bioavailability by 4.7-fold compared with the marketed oral tablet [11]. Kafe et al. prepared niosomes enriched with cetyl alcohol. They stated that cetyl alcohol enhanced the drug encapsulation and served as a stabilizing agent by forming hydrogen bonds with the hydroxyl group of Span 40, preventing aggregation and enhancing stability [12].

A passive targeting strategy has drawbacks, like its low tumor specificity. Hence, various active targeting strategies have been tried to overcome the drawbacks of the passive targeting strategy [13]. Hyaluronic acid (HA) is a crucial component of the extracellular matrix. HA has been extensively utilized in biomedical applications due to its low immunogenicity and toxicity, and excellent biodegradability and biocompatibility [14, 15]. HA is known as an active tumor-targeting ligand for the CD44 receptor, which is overexpressed in many different types of cancers. Thus, HA can actively and selectively deliver anticancer drugs to the desired tumor region, reducing adverse reactions that may happen in normal tissues and organs [16, 17]. In previously published studies, the anticancer efficacy of drugs in different nanoparticle formulations has been examined in multiple cancer cell lines and animal models. Sallam et al. prepared hyaluronic-coated cubosomes for anticancer drug delivery. They stated that HA dramatically enhanced the anticancer effect than the uncoated cubosomes in pancreatic cancer cells [18]. Ma et al. prepared PEGylated hyaluronic acid nano-drug for anticancer drug delivery. They stated that HA succeeded in achieving tumor targetability and showed a strong anticancer effect in colon cancer cells [19].

Hyaluronic acid coated cetylosomes (HCCs) are novel nanocarriers that were prepared in this design to be loaded with FEL to enhance its solubility and investigate its potential anticancer efficacy. HCCs are lipophilic nanoparticles composed of Brij 97, cetyl alcohol (CA), and Hyaluronic acid (HA). Brij 97 was used as a surfactant to boost the drug solubility and dissolution rate. CA is a fatty base that is added to form the bilayer structure that is essential for encapsulating drugs and to impart a lipophilic environment to increase the lipophilic drug’s entrapment efficiency %. Also, CA is metabolized by aldehyde dehydrogenase and alcohol dehydrogenase, which are overexpressed in many cancer cells [20, 21]. Hence, the incorporated cetyl alcohol in the HCCs could target the cancer cells. Moreover, HA was added to coat the prepared nanoparticle to achieve active targeting to the cancer cells by inducing CD44-mediated intracellular signal transmission. So, HCCs could be completely and selectively uptaken by the cancer cells. CA and Brij 97 were chosen as they have excellent biocompatibility in blood [22, 23].

Yet, the nanoparticles are physically unstable due to their aggregation during storage. Hence, converting the nanoparticles to a solid dosage form is a great solution to avoid this issue. Lyophilization, or freeze-drying, is a dehydration technique used to boost the nanoparticles’ stability, extend their shelf-life, and facilitate their handling during storage [24]. The FEL optimized HCC (FEL-OHCC) formula was lyophilized to be reconstituted before parenteral administration.

Considering these facts, it is highly appealing to use the combined benefits of both FEL-HCCs and the parenteral route of administration to potentiate the drug solubility and anticancer efficacy. So far, there is no published paper formed FEL-HCCs to investigate FEL anticancer efficacy, exploring the novelty of this study.

This study aimed to prepare FEL-HCCs to enhance FEL solubility and anticancer efficacy. FEL-HCCs were prepared using thin-film hydration technique adopting Box-Behnken Design. FEL-HCCs were examined by assessing their vesicle size, polydispersity index, zeta potential, and entrapment efficiency %. The formula that showed the highest desirability value was chosen as the FEL optimized HCC (FEL-OHCC) formula. It was lyophilized and sterilized to form a porous powder to be reconstituted before parenteral administration. The lyophilized sterilized OHCC formula was subjected to an in vitro release study and stability study. At the end, cell line and in vivo studies were implemented to assess the anticancer efficacy of the lyophilized, sterilized FEL-OHCC.

## Materials and methods

### Materials

Felodipine (FEL) was provided by Global Napi Pharmaceutical Co. (Cairo, Egypt). Hyaluronic acid (HA) was purchased from Acros Organics, Belgium. Brij 97 and Cetyl alcohol (CA) were purchased from Sigma-Aldrich (St. Louis, USA). FEL oral market tablet (Felodipine STADA 10 mg) was obtained from Global Napi Pharmaceutical Co. (Cairo, Egypt). Chloroform and Methanol were obtained from El-Nasr Pharmaceutical Co. (Cairo, Egypt).

### Box-Behnken experimental design

FEL-hyaluronic acid coated cetylosomes (HCCs) were prepared according to a Box-Behnken experimental design with 3 independent variables at 3 different levels to assess the impact of the studied factors on vesicles’ properties. This design aids in reducing the number of experimental runs, reducing costs, and saving time [11]. Table 1 displays the factors studied, their levels, and constraints. The three independent variables were the different amounts of Brij 97 amount (X_1_), Cetyl alcohol (X_2_), and Hyaluronic acid (X_3_). The entrapment efficiency % (EE%), the vesicle size (VS), the polydispersity index (PDI), and the zeta potential (ZP) were the examined responses. The optimized FEL-HCCs formula was selected based on showing the highest desirability employing Design-Expert^®^ software (Stat-Ease, Inc., Minneapolis, USA).

**Table 1:** Variables and constraints in Box-Behnken experimental design for Optimization of FEL-HCCs

	**Applied Levels**		
	**Low**	**High**	
**Independent Variables**			
X_1_: Brij 97 amount (mg) X_2_: Cetyl alcohol amount (mg) X_3_: Hyaluronic acid amount (mg)	50	75	100
	20	30 7.5	40
	5		10
**Dependent Variables**	**Goal**		
Y_1_: Entrapment Efficiency (EE%)	Maximize		
Y_2_: Vesicle Size (VS) (nm)	Minimize		
Y_3_: Polydispersity index (PDI)	Minimize		
Y_4_: Zeta potential (ZP) (mV)	Maximize (as absolute value)		

### Preparation of FEL-HCCs

FEL-HAECs were prepared utilizing the Thin-film hydration technique [25]. FEL (10 mg), Brij 97, and CA were weighed accurately and dissolved in a 10 mL mixture of methanol:chloroform (1:2v/v) in a 250 mL round bottom flask. The resulting organic solvents were evaporated under vacuum utilizing a rotary evaporator (Heidolph VV 2000, Burladingen, Germany), revolved for 1 h at 150 rpm and 60 ºC. The formed thin film was hydrated utilizing 10 mL of distilled water containing HA. Then the flask was rotated for 1 h under normal pressure with the same fixed speed and temperature. Finally, the resulting dispersions of the FEL-HCCs were sonicated for 3 min to reduce vesicle size.

### Characterization of the prepared FEL-HCCs

#### Determination of FEL-HCCs entrapment efficiency percentage (EE%)

The ultracentrifugation technique was used to detect the EE% of the prepared FEL-HAECs. 3 mL was taken from each FEL-HCC and centrifuged for 1 h at 22,000 rpm and 4 ºC utilizing a cooling centrifuge (Sigma 3 K 30, Germany). Then, an aliquot from the resulting supernatant was taken, properly diluted with methanol, and measured spectrophotometrically (Shimadzu, Kyoto, Japan) at λ_max_ 361 nm to estimate the unentrapped concentration of FEL [4]. The EE% was calculated utilizing the following equation [26]:

1 $$EE\%=\frac{Total \;FEL\;concentration-Concentration\;of \;unentrapped \;FEL}{Concentration\;of\;unentrapped\;FEL}\times100$$

The study was repeated in triplicate, and the mean EE% ± SD was determined.

#### Determination of FEL-HCCs vesicle size (VS), polydispersity index (PDI), and zeta potential (ZP)

The VS, PDI, and ZP of all the prepared FEL-HCCs were assessed by using a Zetasizer (Malvern, Worcestershire, UK) at 25 ºC [27]. PDI is a vital indicator of the prepared FEL-HCCs’ size distribution. ZP was measured to assess the physical stability of the prepared FEL-HCCs. ZP was determined by tracking mobile vesicles in an electric field. The samples taken from each formula were adequately diluted before measurement to reach a suitable light scattering intensity [28]. All measurements were repeated three times, and the averages ± SD were recorded.

### Selection of the optimized HCC (OHCC) formula

Optimization was implemented to conclude the studied factors’ levels (X_1_: Brij 97 amount, X_2_:CA amount, and X_3_: HA amount) by which a maximum value of EE% and ZP (as absolute value) and minimum values of VS and PDI can be achieved by applying the Design Expert^®^ software. For approving the model performance, the OHCC formula was prepared, characterized, and the obtained observed responses were compared to the predicted responses through calculation of the bias percent by utilizing the following equation [29]:

2 $$Basic\;percentage=\frac{\vert Predicted\;value-observed\;value\vert}{observed\;value}\times100$$

### Lyophilization and sterilization of the OHCC formula

The OHAEC formula was subjected to freezing at—20 ºC in the presence of mannitol (2% w/v) as a cryoprotectant. Then, it was lyophilized for 24 h under pressure of 7 × 10^−2^ mbar at—45 ºC (Novalyphe-NL 500; Savant Instruments Corp., USA) [30]. VS, PDI, ZP, and EE% were measured to be statistically compared with the values of the OHCC formula before lyophilization to detect the impact of lyophilization on the properties of the optimized formula.

After that, to sterilize the lyophilized OHCC (LY-OHCC) formula, the lyophilized OHCC formula was packed in dry ice to evade the possible melting or aggregation caused by the gamma radiation-induced temperature elevation (Gamma cell 1000, Ontario, Canada) [30]. The dose of radiation was 2.5 Mrad as declared in the United States pharmacopoeia [31]. Then, the sterilized LY-OHCC formula was reconstituted with water for injection, and VS, PDI, ZP, and EE% were remeasured and compared with the LY-OHCC formula’s results before sterilization to detect the impact of sterilization on the properties of the LY-optimized formula. Data was statistically analyzed and considered different at p-values < 0.05.

### Characterization of the sterilized LY-OHCC formula

#### Drug content (DC)

0.1 gm from the sterilized LY-OHACC formula was dissolved in 10 ml of methanol, followed by sonication for 1 min to assure the disruption of all vesicles. The resulting clear solution was measured spectrophotometrically in triplicate at λ_max_ 361 nm (Shimadzu, Kyoto, Japan) [4].

#### Transmission electron microscope (TEM)

The morphological evaluation of the OHCC formula and the sterilized LY-OHCC formula was implemented utilizing a TEM (Joel JEM 1400, Tokyo, Japan) to assess the impact of lyophilization and sterilization on the vesicle size of the OHCC formula. Briefly, samples from the examined formulations were diluted utilizing distilled water, then placed on copper plates coated with carbon, dyed with 2% phosphotungstic acid, left to dry at 25 ºC, and then examined at 100 kV [25, 32].

#### Scanning electron microscopy (SEM)

SEM (JXA-840; JEOL, Japan) was utilized to examine the morphological structure of the LY-OHCC formula’s surface before and after sterilization. Examination was conducted after coating the LY-OHCC formula and the sterilized LY-OHCC formula with gold under vacuum [30].

#### In vitro FEL release study

In vitro FEL release from the OHCC formula before and after lyophilization and sterilization was carried out utilizing the membrane diffusion technique in modified USP dissolution apparatus I (Pharm Test, Hainburg, Germany). Before starting the study, the LY-OHCC formulations before and after sterilization were reconstituted utilizing distilled water. A sample from each formula equivalent to 5 mg FEL was supplied to a plastic cylinder covered with the dialysis membrane (12–14 kDa molecular mass cut-off) from one end. The cylinder’s other end was fixed to the dissolution apparatus’s shaft. The receiving medium was 500 mL phosphate buffer saline (pH = 7.4) [33–36]. This study was carried out without adding sink conditions enhancers to determine the impact of the nano formula alone on enhancing the drug release rate and extent [37]. The shaft’s rotation speed was adjusted to 50 rpm, and the temperature of the receiving media was fixed to 37 ºC during the study. The release study of the FEL oral market tablet was implemented under the same conditions. 3 mL samples were gathered at different time intervals of 0.25, 0.5, 1, 2, 3, 4, 5, 8, 12, and 24, and analyzed by UV-spectrophotometry at λ_max_ 361 [4]. The test was repeated in triplicate, and the average released percentages ± SD were recorded. Similarity factors (*f2*) and half-lives were calculated for comparison of release profiles. The mechanism of FEL release from the OHCC formula before and after lyophilization and sterilization was detected by subjecting the release data to different kinetic models (zero order, first order, Higuchi, and Korsmeyer-Peppas model equations. The correlation coefficient (R^2^) was estimated for each model, and the highest R^2^ value indicated the best-fit model to the mathematical equation [25].

#### Stability study

The sterilized LY-OHCC’s stability was investigated by storing it in a well-closed glass container and keeping it for 6 months at room temperature. The mean DC, VS, PDI, ZP, and EE% were assessed and compared to the fresh sterilized LY-OHCC formula’s results before storage [26].

### In vitro cytotoxicity study using human breast adenocarcinoma cell line (MCF-7)

#### MTT assay

The MCF-7 cell line, a human breast adenocarcinoma cell line, was sourced from the American Type Culture Collection (ATCC, USA). Cells were maintained in Dulbecco’s Modified Eagle’s Medium (DMEM) supplemented with 10% fetal bovine serum (FBS) and 1% penicillin–streptomycin and cultured at 37 °C in a humidified 5% CO₂ incubator.

Cells were seeded into 96-well plates (1.0 × 10^3^ cells/well) and treated with varying concentrations of doxorubicin (DOX), felodipine (FEL), or OHCC for 48 h. Following treatment, cell viability was determined using the MTT assay (3-(4,5-dimethylthiazol-2-yl)−2,5-diphenyltetrazolium bromide). The percentage of cell viability (CV%) was calculated as [38, 39]:

3 $$CV\%\;=\frac{\text{Absorbance of treated cells }}{\text{Absorbance of control cells }}\mathrm{x}100$$

GraphPad Prism version 9 was utilized to generate dose–response curves and compute IC₅₀ values.

#### Trypan blue assay for evaluating cancer cell growth inhibition

The trypan blue assay was carried out to evaluate cell growth inhibition by DOX and/or OHCC treatment. Six-well plates were seeded with 1 × 10^6^ cells per well and incubated overnight at 37 °C and 5% CO₂. Then, cells were treated with either half the IC_50_ concentration of DOX and/or the OHCC using two different concentrations and then incubated for 48 h. Cells were then collected by trypsinization and washed twice with PBS. After staining with trypan blue, viable and non-viable cells were counted using a hemocytometer [40].

#### Fluorescent stain for evaluating the mechanism of cell death

Acridine orange/ethidium bromide (AO/EB) dual fluorescent staining was employed to evaluate cell death. MCF-7 cells were exposed to half the IC₅₀ concentrations of DOX, OHCC, or their combination for 24 h. Cells were then harvested to be stained with AO/EB solution (1:1 ratio) and immediately examined under a fluorescence microscope [41].

For each treatment group, at least 500 cells were counted. Viable cells displayed green fluorescence, apoptotic cells exhibited yellow to orange staining, while necrotic cells showed dark orange to red fluorescence. The apoptotic and necrotic populations were calculated relative to the total number of counted cells.

#### Cell pellet preparation and ELISA assay

To investigate the protein expression levels of apoptosis- and angiogenesis-related markers, an ELISA assay was performed targeting Bax, Bcl-2, caspase-9, and vascular endothelial growth factor (VEGF). Bax (a pro-apoptotic protein) and Bcl-2 (an anti-apoptotic protein) are key regulators of the mitochondrial apoptotic pathway [42, 43], while caspase-9 functions as an initiator caspase in apoptosis [44]. VEGF, on the other hand, promotes angiogenesis, which plays a central role in tumor growth and progression [45]. Therefore, antiangiogenic therapy represents a promising treatment strategy.

Cells were treated with DOX (0.83 µg/ml; half IC₅₀) and/or OHCC at two concentrations (IC₅₀ and half IC₅₀, 5 and 2.5 µg/ml, respectively) for 48 h. Following treatment, cells were collected to prepare cell lysates using ice-cold lysis buffer. The lysates were used for ELISA according to the kit manufacturer’s protocol (Sunlong Biotech, China). Absorbance values were measured, and protein concentrations were quantified using an ELISA reader (FLUOStar Omega, BMG Labtech, Germany).

### In vivo studies

#### Animals

Female albino mice (10–12 weeks, 25–35 g) were obtained from the animal facility at Cairo University, Faculty of Veterinary Medicine. The mice were housed in plastic cages with unrestricted access to food and water at 20 ±  2 °C, 55 ± 5% relative humidity, and a 12 h light/dark cycle. Experiments complied with NIH guidelines and were approved by the Institutional Animal Care and Use Committee (protocol PT 3742).

Ehrlich ascites carcinoma (EAC) cells were maintained in male BALB/c mice [46]. Cells were serially passaged according to standard procedures. Briefly, ascitic fluid was withdrawn, diluted with sterile PBS (1 ml), and injected intraperitoneally into BALB/c mice. After 7 days, 200 μl of the diluted suspension was injected intraperitoneally into 4–6-week-old male BALB/c mice for tumor propagation [47].

#### Induction of the solid form of Ehrlich carcinoma (ESC) and experimental design

To establish solid tumors, mice were injected subcutaneously in the left thigh with EAC cells (Day 0). On Day 10 post-inoculation, the animals were randomly divided into seven experimental groups (n = 10 per group):

- **Group I (negative control):** Mice treated with normal saline intraperitoneally (i.p.).
- **Group II (positive control):** ESC-bearing mice treated with normal saline intraperitoneally (i.p.).
- **Group III (DOX):** Ehrlich-bearing mice treated with 2.5 mg/kg/2 days doxorubicin in six equal i.p. injections for two weeks for a total cumulative dose of 15 mg/kg [48, 49]
- **Group IV (FEL):** Ehrlich-bearing mice treated with 5 mg/kg/day pure felodipine i.p. [50, 51].
- **Group V** (**Blank formula**)**:** Ehrlich-bearing mice treated with 5 mg/kg/day blank formula i.p.
- **Group VI (FEL-OHCC):** Ehrlich-bearing mice treated with 5 mg/kg/day felodipine optimized hyaluronic acid-coated cetylosomal formula i.p.
- **Group VII** (**DOX + FEL-OHCC):** Ehrlich-bearing mice treated with 2.5 mg/kg/2 days DOX and 5 mg/kg/day FEL-OHCC formula i.p.

The treatment continued for 15 consecutive days.

#### Assessment of tumor volume, growth inhibition rate, and tumor weight

Every day, mice were closely monitored. Two-dimensional measurements with Vernier callipers were used to evaluate tumor growth, and measurements were taken every three days. The ellipsoidal tumor volume (V) was measured and estimated using the formula [52]:

V = (D*d)^2^/2, where D and d, are the long and short axes, respectively.

TIR% (tumor-growth inhibition rate) was measured, which is equal to (C − T)/C × 100, where T is the treatment group's mean tumor volume and C is the control group's mean tumor volume. For measurement of the tumor weight, the whole tumor area was separated from the thigh at the end of the experiment and weighed.

#### Determination of the survival time, percent increase in life span

The animals were observed for their mortality daily until the end of the experiment. The animals' spontaneous deaths served as the experiment's endpoint, and the mean survival time (MST) was computed using the formula MST = (day of first death + day of last death)/2. Using the formula of Nicol and Prasad [53]: (ILS% = (T − C)/C × 100), where T is the MST of the treated animals, and C is the MST of the control group, the percentage of increase in life span (ILS) was determined. MST of treated animals divided by MST of the control group was used to compute T/C% (treated vs. Ehrlich control). The National Cancer Institute's (NCI) standards state that a medication has significant antitumor efficacy if its T/C and ILS are greater than 125% and 25%, respectively [54].

#### Histopathological examination

Histopathological investigations involved immediate immersion of fresh muscle tissue samples in a 10% buffered formalin solution. The fixed tissues were soaked in paraffin wax and subsequently sectioned into thin sections with an average thickness of 5 µm using a microtome. The sections were then stained with hematoxylin and eosin dyes. The stained slices were captured in photographs and inspected using a light microscope.

#### Statistical analysis

Every result is displayed as a mean ± SD. GraphPad Prism software version 9 (GraphPad Software, San Diego, USA) was used for all statistical calculations. One-way ANOVA followed by Tukey multiple comparison test as a post hoc test was used for the analysis of data. Differences at *p* < 0.05 were considered significant.

## Results and discussion

### Statistical analysis of experimental design

**Fig. 1:**
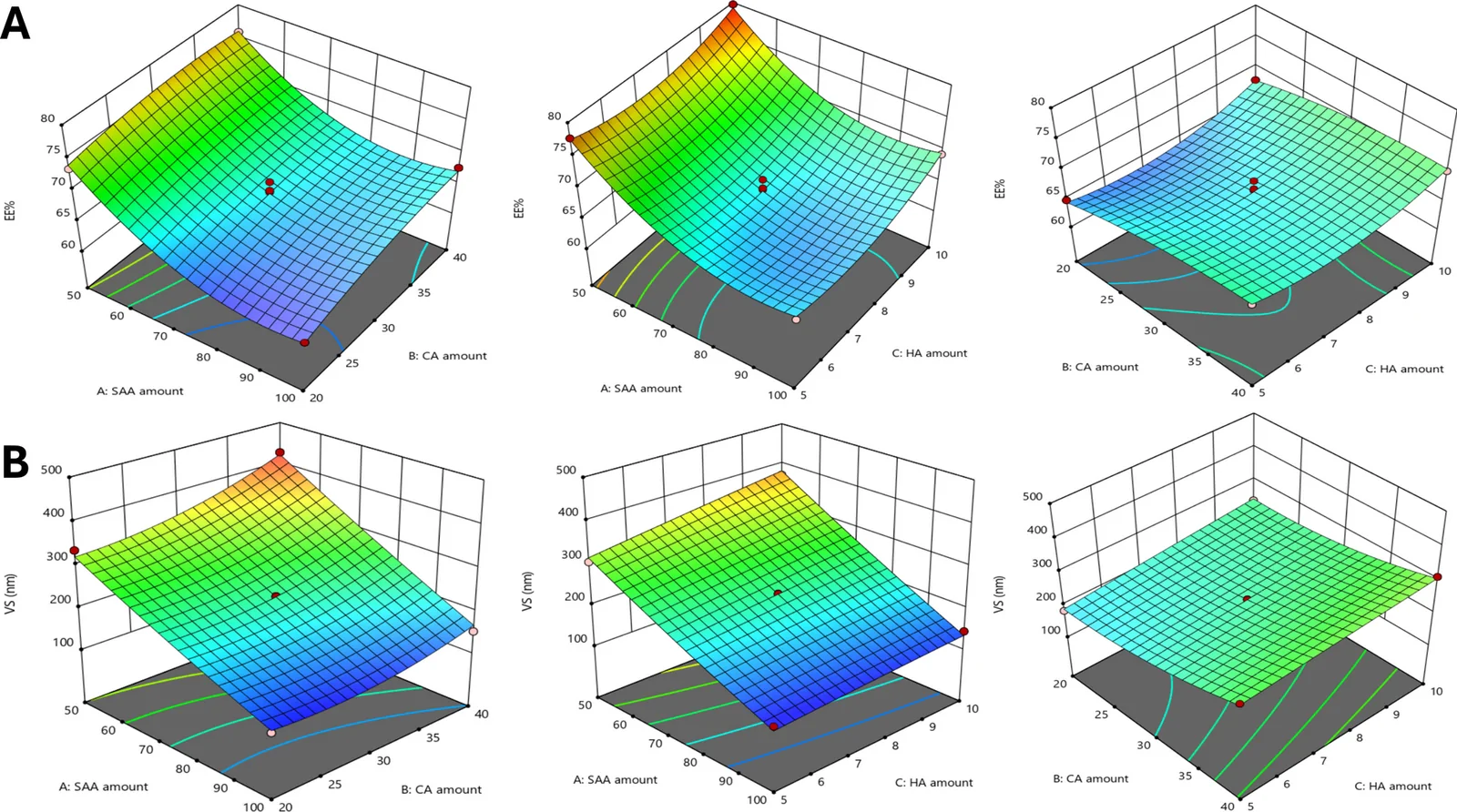
3D surface plot showing the effect SAA Amount (X_1_), CA amount (X_2_), and HA amount (X_3_) on Entrapment Efficiency *%* (**A**) and Vesicle Size (**B**) of FEL-HCCs

**Fig. 2:**
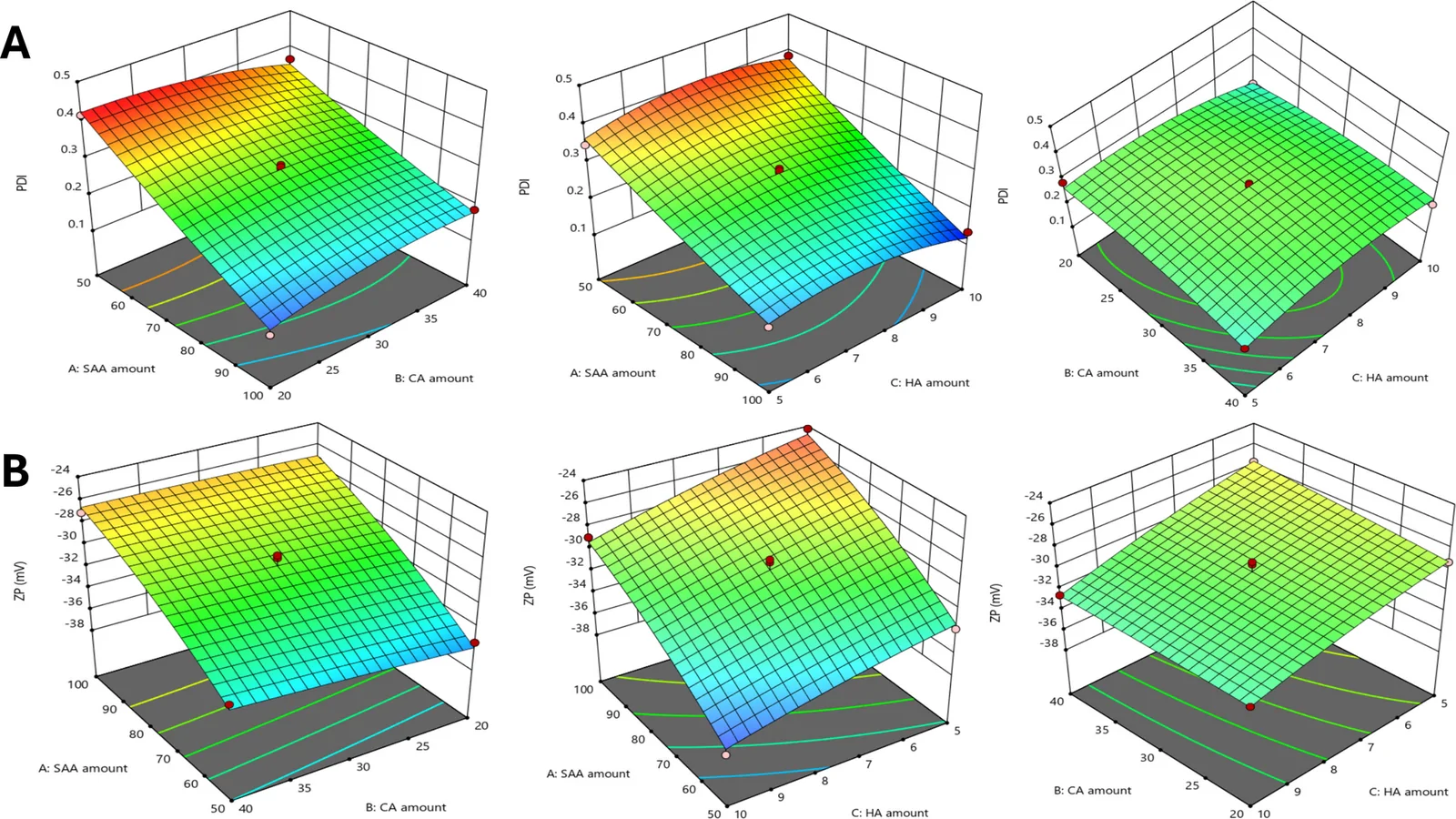
3D surface plot showing the effect SAA Amount (X_1_), CA amount (X_2_), and HA amount (X_3_) on Polydispersity index (**A**) and Zeta potential (**B**) of FEL-HCCs

Box-Behnken experimental design was employed to investigate different variables at different levels using Design Expert software. Three factors at three levels were studied in this study, and four different responses were measured, as displayed in Table 1. Table 3 details the output data of the experimental design. The values of adequate precision were higher than 4 in all the dependent variables, which confirms the suitability of the employed model. Moreover, the predicted and adjusted R^2^ values aligned well in all the examined dependent variables, affirming the capability of the utilized model to navigate the design space for prediction and optimization. Figure 1 and 2 showed the 3D response surface plot diagrams that illustrated the effect of the independent variables on the studied dependent variables.

### Characterization of the prepared FEL-HCCs

#### Determination of FEL-HCCs Entrapment efficiency percentage (EE%)

EE% is a beneficial parameter to evaluate the HCCs to obtain optimal drug encapsulation efficiency. The high EE% values indicate that FEL-HCCs can offer an efficient delivery system for poorly water-soluble drugs such as FEL. The EE% of FEL in the prepared HCCs ranged from 62.12 ± 3.14% to 79.74 ± 5.72%, as displayed in Table 2. The equation used to assess the EE% results was:

4 $$\mathrm{EE\%}=66.51- 5.26*{\mathrm{X}}_{1}+1.78*{\mathrm{X}}_{2}+1.31*{\mathrm{X}}_{3}+ 0.76*{\mathrm{X}}_{1}*{\mathrm{X}}_{2}+ 0.45*{\mathrm{X}}_{1}*{\mathrm{X}}_{3} - 0.36*{\mathrm{X}}_{2}*{\mathrm{X}}_{3} + 4.25* {\mathrm{X}}_{1}^{2} - 0.98*{\mathrm{X}}_{2}^{2} + 2.35*{\mathrm{X}}_{3}^{2}$$

**Table 2:** Experimental runs, independent variables, and measured responses of FEL-HCCs

F	X_1_: Brij 97 amount (mg)	X_2_: CA amount (mg)	X_3_: HA amount (mg)	Y_1_: EE%	Y_2_: VS (nm)	Y_3_: PDI	Y_4_: ZP (mV) (absolute value)
1	50	30	10	79.74 ± 5.72	371.9 ± 11.35	0.387 ± 0.03	−37.91 ± 2.26
2	50	30	5	77.71 ± 3.62	304.34 ± 8.42	0.347 ± 0.05	−33.75 ± 1.54
3	50	20	7.5	73.45 ± 4.17	335.61 ± 10.63	0.452 ± 0.03	−35.42 ± 3.67
4	50	40	7.5	75.93 ± 2.35	427.47 ± 9.38	0.368 ± 0.04	−33.9 ± 1.27
5	75	40	5	68.64 ± 2.08	275.26 ± 8.43	0.206 ± 0.02	−27.66 ± 2.46
6	75	30	7.5	66.97 ± 3.68	230.56 ± 5.41	0.291 ± 0.03	−30.28 ± 1.58
7	75	30	7.5	65.38 ± 4.47	232.86 ± 7.12	0.264 ± 0.05	−30.93 ± 3.04
8	75	20	10	67.83 ± 2.85	237.98 ± 9.64	0.199 ± 0.01	−32.89 ± 2.64
9	75	30	7.5	66.74 ± 1.97	231.04 ± 14.39	0.282 ± 0.03	−29.53 ± 1.96
10	75	30	7.5	68.37 ± 3.15	228.36 ± 12.55	0.297 ± 0.02	−31.02 ± 2.38
11	75	20	5	64.81 ± 2.28	183.82 ± 8.28	0.287 ± 0.04	−29.23 ± 1.14
12	75	40	10	70.21 ± 4.06	312.17 ± 13.25	0.221 ± 0.03	−32.54 ± 4.03
13	75	30	7.5	65.08 ± 2.35	230.88 ± 7.28	0.286 ± 0.02	−29.28 ± 3.44
14	100	30	10	69.43 ± 1.58	143.53 ± 10.37	0.134 ± 0.01	−28.94 ± 2.16
15	100	20	7.5	62.12 ± 3.14	118.72 ± 6.15	0.114 ± 0.02	−27.46 ± 1.28
16	100	40	7.5	67.64 ± 2.94	151.89 ± 12.46	0.186 ± 0.02	−27.12 ± 1.49
17	100	30	5	65.58 ± 3.42	120.41 ± 5.68	0.147 ± 0.03	−24.23 ± 1.43

All the formulated FEL-HCCs included equal amounts of FEL (10 mg). Data are presented as mean ± SD (n = 3). FEL: Felodipine, F: formula, CA: cetyl alcohol, HA: hyaluronic acid, EE%: entrapment efficiency percent, VS: vesicle size, PDI: polydispersity index, and ZP: zeta potential.

From Fig.  1 A, the EE% was significantly impacted by all the studied factors: Brij 97 amount (X_1_), Cetyl alcohol amount (X_2_), and Hyaluronic acid amount (X_3_), with P values of < 0.0001, 0.0027, and 0.0126, respectively.

Increasing the surfactant (Brij 97) amount (X_1_) had a significant negative impact on entrapping the drug within the vesicles of the prepared HCCs, as shown in Fig. 1A. The EE% of the prepared FEL-HCCs significantly decreased with increasing the Brij 97 amount from 50 to 100 mg. This was attributed to increasing the fluidity of the bilayers’ membrane and creating highly permeable pores in the vesicle bilayer by increasing the amounts of Brij 97, thus resulting in increased drug leakage and decreasing EE% values [55]. Similar findings were stated by Deng et al., who prepared PEGylated ultra-deformable transferosomes loaded with tapentadol. They stated that increasing the Brij amount significantly decreased the EE of the drug within the prepared PEGylated transferosomes [56].

On the other hand, increasing the CA amount (X_2_) had a significant positive effect on the EE% values. Increasing the CA amount efficiently increased the encapsulation capacity due to increasing CA’s alkyl chains, which form strong hydrophobic bonds with the hydrophobic drug, boosting FEL accommodation within the vesicles’ bilayer [11]. Additionally, increasing the CA amount increased the lipophilic environment, hence enhancing the hydrophobic drug’s encapsulation [39]. These results agreed with Vankayala et al., who formed cetyl alcohol based novel nanovesicles loaded with resveratrol, and stated that increasing the CA amount remarkably enhanced the drug entrapment efficiency % [57].

Regarding increasing the HA amount (X_3_), it was observed to significantly increase the EE% of FEL within the formed FEL-HCCs. This could be due to increasing the formulation’s viscosity upon increasing the HA amount, leading to hindrance of drug diffusion and ensuring its stay entrapped within the formed vesicles [29]. Additionally, this prevented the drug leakage from the prepared HCCs [58]. These results agree with Sadeghi-Ghadi et al., who prepared hyaluronic acid enriched niosomes loaded with quercetin. They stated that increasing the HA amount in the niosomal formulation significantly increased quercetin EE% within the niosomal vesicles [59].

#### Determination of FEL-HCCs vesicle size (VS)

Vesicle size is an important factor in many aspects. The prepared FEL-HCCs had VS values ranging from 118.72 ± 6.15 to 427.47 ± 9.38 nm, as displayed in Table 2. The equation utilized to assess the VS results was:

5 $$\mathrm{VS}=230.74-113.01*{\mathrm{X}}_{1}+36.33*{\mathrm{X}}_{2}+22.71*{\mathrm{X}}_{3}-14.67*{\mathrm{X}}_{1}*{\mathrm{X}}_{2}-11.11*{\mathrm{X}}_{1}*{\mathrm{X}}_{3}-4.31*{\mathrm{X}}_{2}*{\mathrm{X}}_{3} + 5.21*{\mathrm{X}}_{1}^{2} + 22.47*{\mathrm{X}}_{2}^{2} - 0.90*{\mathrm{X}}_{3}^{2}$$

From Fig. 1B, the EE% was significantly affected by all the factors studied: Brij 97 amount (X_1_), Cetyl alcohol amount (X_2_), and Hyaluronic acid amount (X_3_), with P values of < 0.0001, < 0.0001, and 0.0011, respectively.

Increasing the Brij 97 amount (X_1_) significantly decreased the VS of the FEL-HCCs, as shown in Fig. 1B. This could be attributed to the surface-active property of Brij 97 which decreased the system’s interfacial tension and augmented the curvature of the resulted vesicles, hence decreasing their VS. Whereas the low amount of Brij might be insufficient to reduce the surface tension and cover the lipids efficiently, and hence, larger vesicles were formed [60]. These results agreed with Albash et al., who prepared fenticonazole nitrate loaded PEGylated cerosomes. They stated that the VS of the formed cerosomes was significantly decreased upon increasing the Brij amount [61].

On the other hand, increasing the CA amount (X_2_) significantly increased the VS of FEL-HCCs. This might be attributed to boosting the aggregation of the formed nanovesicles upon increasing the level of lipids. Hao and li stated similar outcomes when they prepared niosomal nanovesicles and observed that increasing the lipid levels led to a significant increase in the niosomal VS [62]. This could also be because increasing the chain length of fatty alcohols increased the lipophilicity, lowering hydrophilic lipophilic balance (HLB) and a higher critical packing parameter, increasing the particle size [10]. These results agreed with Vankayala et al., who prepared nanovesicles using different types of fatty alcohols as lipid components, and reported that the particle size of the vesicles increased significantly with increasing the amount of the fatty alcohol [57].

There was a significant interaction between the Brij 97 amount (X_1_) and the CA amount (X_2_) (*p* = 0.0453), as shown in supplementary Fig SF1. Where at low level of surfactant (Brij 97) amount, the high level of CA significantly formed vesicles with large PS, while at the high level of surfactant amount, increasing the CA amount to the high level didn’t significantly enlarge the VS. Where the VS of the FEL-HCCs was small at the high level of Brij 97 weather at high or low levels of CA. This could be attributed to increasing the surfactant (Brij 97) concentration to the highest level, which increased the solubilization of FEL in the fatty alcohol (CA), resulting in a higher velocity of coverage of the formed nanoparticle surfaces and packing properties of the surfactants, preventing aggregation of nanovesicles despite high levels of CA [10]. This also could be attributed to the high flexibility of the surfactant (Brij 97) layer and its ability to partition at higher levels into the lipid-water interface, which stabilized the formed nanovesicles [63].

Furthermore, increasing the HA amount (X_3_) was observed to significantly increase the VS of the FEL-HCCs. This could be attributed to the HA’s adsorption on the vesicles’ wall, forming a coat that increased the VS of the FEL-HCCs [64]. This could also be because HA is a hydrophilic molecule that can absorb water and swell in aqueous conditions, increasing its size and hence increasing the VS of HCCs [65]. These results agreed with Fahmy et al., who prepared hyaluronic acid-enriched ultradeformable elastosomes loaded with voriconazole. They stated that increasing the HA amount markedly increased the VS of the prepared elastosomes [66].

#### Determination of FEL-HCCs poly dispersity index (PDI)

PDI value is considered indicative of the uniformity of the nanoparticles’ distribution. The ideal PDI value is less than 0.3. As displayed in Table 2, PDI values of the prepared FEL-HCCs ranged from 0.114 ± 0.02 to 0.452 ± 0.03, reflecting the uniformity of the size of the prepared HCCs. The equation utilized to assess the PDI results was:

6 $$\mathrm{PDI}=0.29-0.12*{\mathrm{X}}_{1}-0.004*{\mathrm{X}}_{2} -0.006*{\mathrm{X}}_{3}+0.03*{\mathrm{X}}_{1}*{\mathrm{X}}_{2}-0.013*{\mathrm{X}}_{1}*{\mathrm{X}}_{3}+ 0.026*{\mathrm{X}}_{2}*{\mathrm{X}}_{3}+ 0.01*{\mathrm{X}}_{1}^{2}-0.02*{\mathrm{X}}_{2}^{2} - 0.04*{\mathrm{X}}_{3}^{2}$$

From Fig.  2 A, PDI values were significantly impacted only by the Brij 97 amount (X_1_) (*P* < 0.0001). While Cetyl alcohol amount (X_2_) (*P* = 0.5539) and Hyaluronic acid amount (X_3_) (*P* = 0.4275) had no significant impact on PDI values.

Increasing the Brij amount (X_1_) significantly decreased the PDI values of the prepared FEL-HCCs, as shown in Fig. 2A. This could be attributed to the efficiency of Brij 97 as a surfactant to prevent the formed nanovesicles’ aggregation, hence decreasing the PDI [67]. This is in good agreement with Albash et al., who prepared PEGylated cerosomes loaded with fenticonazole nitrate. They stated that increasing the Brij amount led to a significant decrease in the PDI values of the formed cerosomes [61].

Moreover, there was significant interaction between the Brij 97 amount (X_1_) and the CA amount (X_2_) (*p* = 0.0156), as shown in supplementary Fig SF2. At the low level of surfactant (Brij 97) amount, the high level of CA significantly formed FEL-HCCs formulations with high PDI values, while at the high level of Brij 97 amount, increasing the CA amount to the high level didn’t increase the PDI value of the formed formulations. Where the PDI values were small at high levels of Brij 97 weather at high or low levels of CA. This could be attributed to the direct relation between the PDI values and the PS, as the smallest PS was detected in FEL-HCCs prepared with the highest level of Brij 97, resulting in a highly uniform distribution for the PS (lower PDI) compared to FEL-HCCs prepared with a low Brij 97 level [68]. These results are supported by Osman et al., who stated that smaller particles tend to display higher uniformity and lower PDI values [69]. This also could be due to the great efficiency of Brij 97 as a surfactant at its high level to prevent the formed nanovesicles’ aggregation despite the high level of CA, hence decreasing the PDI [67].

#### Determination of FEL-HCCs zeta potential (ZP)

ZP values reflect the surface charge of the prepared HCCs. The high ZP values (± 20–30 mV) are very important to prevent the vesicles’ aggregation and assure their electrostatic stabilization. ZP values of HCCs ranged from—24.23 ± 1.43 to—37.91 ± 2.26 mV, as displayed in Table 2. The equation utilized to assess the ZP results was:

7 $$\mathrm{ZP}=-30.21+4.73\ast{\mathrm{X}}_1+0.47\ast{\mathrm{X}}_2\mathrm{-}2.18\ast{\mathrm{X}}_3\mathrm{-}0.29\ast{\mathrm{X}}_1\ast{\mathrm{X}}_2-0.14\ast{\mathrm{X}}_1\ast{\mathrm{X}}_3-0.31\ast\mathrm{X}2\ast{\mathrm{X}}_3-0.70\ast\mathrm{X}_1^2-0.07\ast\mathrm{X}_2^2-0.30\ast\mathrm{X}_3^2$$

From Fig. 2B, ZP values were significantly affected by the Brij 97 amount (X_1_) (*P* < 0.0001) and the Hyaluronic acid amount (X_3_) (*P* = 0.0001). While the change in Cetyl alcohol amount (X_2_) had no significant impact on ZP values (*P* = 0.1311).

Increasing the Brij amount (X_1_) led to a significant decrease in ZP values of the FEL-HCCs, as shown in Fig. 2 B. This could be attributed to the presence of a high number of hydrophilic PEG units in Brij 97 (10 units) that formed a steric barrier that screened the charges at the vesicles’ surface, lowering the ZP values [61]. This is assured by Albash et a., who prepared trans-novasomes loaded with Fenticonazole nitrate, and stated that increasing the Brij amount led to a significant decrease in ZP values [70].

On the other hand, increasing the HA amount (X_3_) significantly increased the ZP values of FEL-HCCs. This could be related to possessing HA on negative surface charges that contributed to increasing ZP values. This result is supported by Tran et al., who prepared Hyaluronic acid-coated solid lipid nanoparticles loaded with vorinostat, and they stated that increasing the HA amount significantly increased the negative ZP values on the vesicles’ surface [71].

### Selection of FEL optimized HCC (OHCC) formula

Using the Design-Expert-7^®^ software, the estimated desirability value was used to choose the optimized HCC formula to find the optimized formula that would achieve all the desired responses at once. The OHCC formula that showed the highest desirability value (0.626) was composed of 100 mg Brij 97, 32.44 mg CA, and 10 mg HA. This OHCC formula succeeded in attaining the desired responses collectively, as it showed the minimum VS (146.72 nm) and PDI (0.131), and the maximum EE% (72.04%) and ZP (−30.58 mV) (as absolute value). As detailed in Table 3, these observed results showed high similarity with the results predicted by the statistical design, assuring the employed model accuracy. The bias percentage was 2.78, 5.05, 3.82, and 3.856% for EE %, VS, PDI, and ZP, respectively. Hence, this low bias percent values reveal the design’s validity to predict the responses [72].

**Table 3:** Output data of the Box-Behnken design and predicted and observed values for the OHCC formula

Responses	EE (%)	VS (nm)	PDI	ZP (mV) (absolute value)
Minimum	62.12 ± 3.14	118.72 ± 6.15	0.114 ± 0.02	−24.23 ± 1.43
Maximum	79.74 ± 5.72	427.47 ± 9.38	0.452 ± 0.03	−37.91 ± 2.26
F value	33.24	92.09	41.00	32.92
p-value	< 0.0001	< 0.0001	< 0.0001	< 0.0001
Adequate precision	20.22	33.72	21.47	21.11
Adjusted R^2^	0.9477	0.9809	0.9524	0.9472
Predicted R^2^	0.9031	0.8672	0.8059	0.8259
R^2^	0.9771	0.9916	0.9762	0.9769
Model	Quadratic	Quadratic	Quadratic	Quadratic
Significant factors	X_1_, X_2_, and X_3_	X_1_, X_2_, X_3_, and X_1_X_2_	X_1_ and X_1_X_2_	X_1_ and X_3_
Observed values of OHCC formula	72.04	146.72	0.131	−30.58
Predicted values of OHCC formula	70.09	139.31	0.126	−29.40

### Formulation of sterilized LY-OHCC formula

The implemented freeze-drying technique successfully evaporated the liquid phase from the OHCC formula and converted it into a powder with a highly porous structure, predicting the stability of the LY-OHCC formula. The mean VS, PDI, ZP, and EE% of the reconstituted LY- OHCC formula were 160.13 ± 4.52 nm, 0.168 ± 0.02, −32.08 ± 2.41 mV, and 71.47 ± 3.18%, respectively. These results had no significant differences from the results of the OHCC formula before lyophilization (p-value > 0.05). This could be due to the presence of the hydrophilic PEG chains of Brij SAA that provided a steric barrier, preventing aggregation and stabilizing the LY-OHCC. These results were in harmony with the previous observations of ELhabal et al., who prepared fluocinolone acetonide loaded nanofibers and demonstrated that it could be sterically lyophilized by incorporation of Brij as pegylated surfactant [65].

Moreover, the sterilization process using gamma radiation didn’t affect the structure of the formed lyophilized powder. After sterilization, there was no significant difference between the remeasured parameters of the sterilized LY-OHCC formula compared to the results of the LY-OHCC formula before lyophilization, nor before sterilization (p-value > 0.05). The mean PS, PDI, ZP, and EE% of the reconstituted sterilized formula was found to be 157.08 ± 5.12 nm, 0.184 ± 0.03, −31.92 ± 3.21 mV, and 70.15 ± 4.66%. respectively. Similar findings were reported by El-Dahmy et al., who prepared lyophilized, sterilized mixed micelles and reported that the sterilization process by gamma radiation didn’t affect the characteristics of the lyophilized optimized mixed micelles formula [30].

### Characterization of the sterilized LY-OHCC formula

#### Drug content (DC)

FEL content in the sterilized LY-OHCC formula was found to be 96.98 ± 3.17%, which complies with the pharmacopeial limit [73]. This finding confirmed that the lyophilization and sterilization process had no significant effect on the drug content of the OHCC formula.

#### Transmission electron microscope (TEM)

**Fig. 3:**
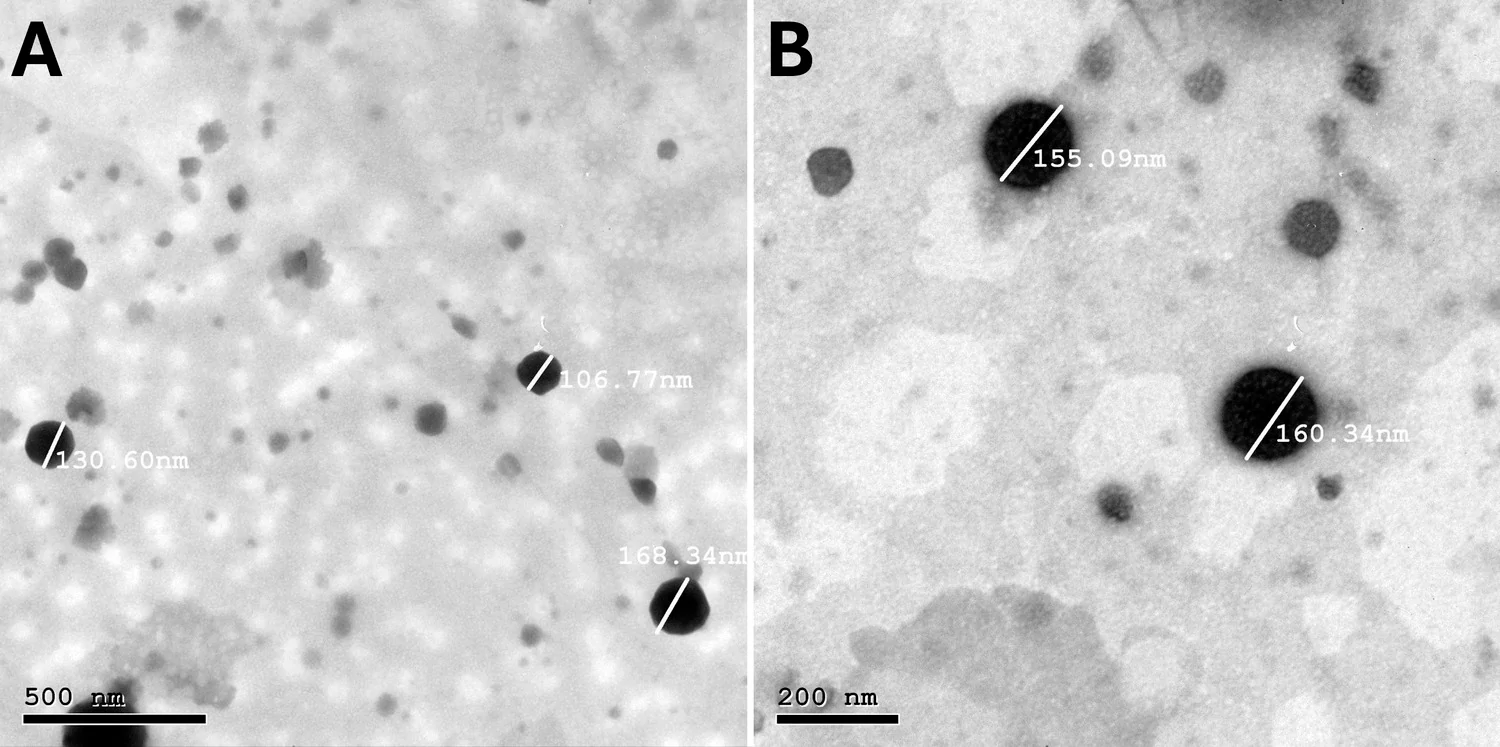
Transmission electron micrographs of (**A**) the OHCC formula and (**B**) the sterilized LY-OHCC formula

TEM micrographs clarified the morphological characteristics of the OHCC formula and the sterilized LY-OHCC formula, as displayed in Fig.  3 A and B, respectively. The micrographs showed spherical-shaped vesicles with a smooth surface and uniform distribution. The average PS of the OHCC and the sterilized LY-OHCC formula detected by the TEM were in the same range, and they closely matched the PS values assessed by the Zetasizer, confirming that lyophilization and sterilization had no significant impact on the OHCC’s vesicles. These findings agree with Fonte et al., who formed lyophilized polymeric nanoparticles. They declared that using the lyophilization technique had no significant effect on the VS of the polymeric nanoparticle, as it prevented their aggregation and maintained their stability [74].

#### Scanning electron microscope (SEM)

**Fig. 4:**
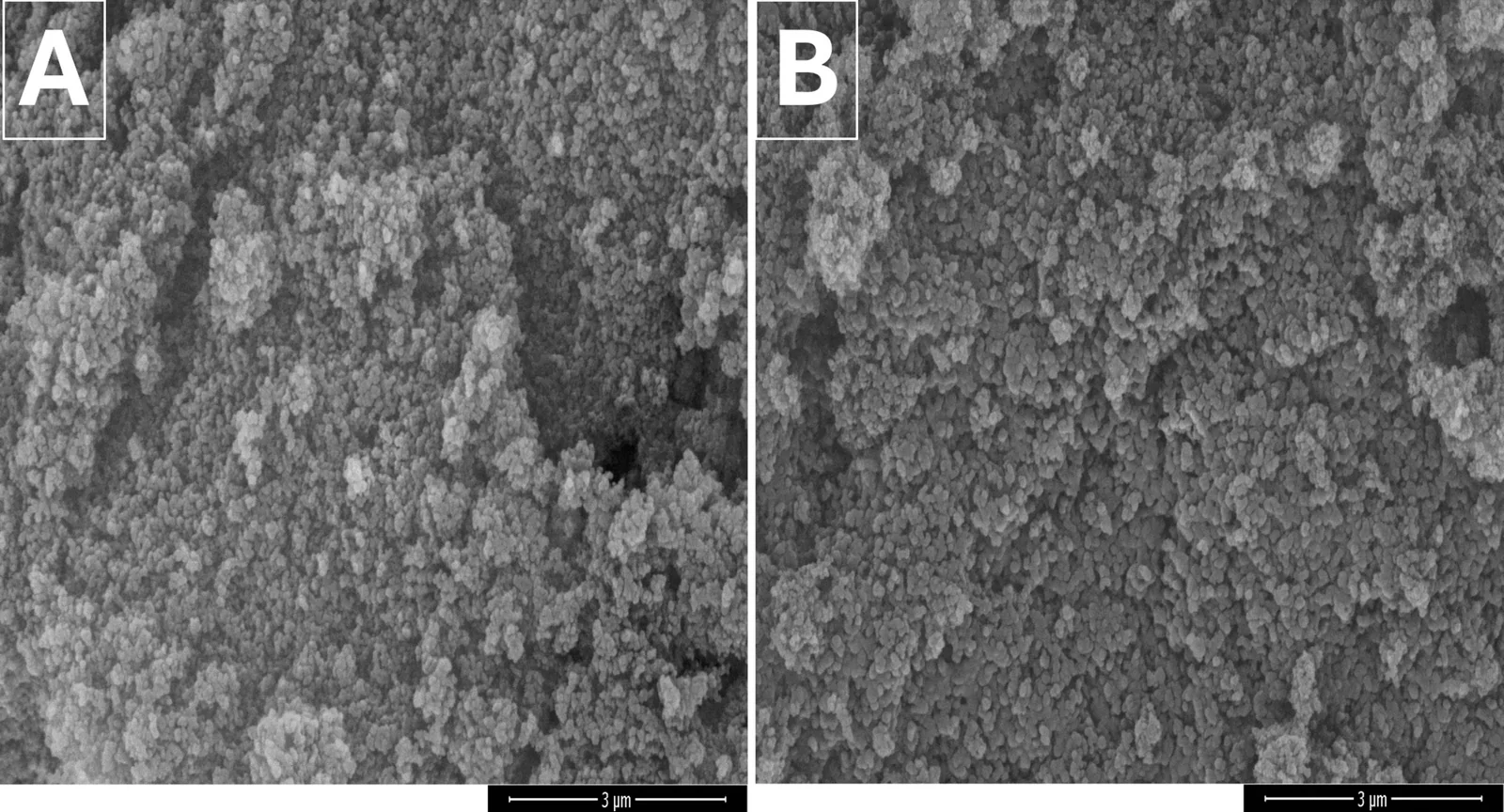
Scanning electron micrographs of the LY-OHCC formula before (**A**) and after (**B**) sterilization

Figure  4 A and B show the SEM micrographs of the LY-OHCC formula before and after sterilization, respectively. Both figures revealed the porous nature of the matrices examined without any significant differences. This reveals that the sterilization process didn’t affect the porous surface nature of the lyophilized powder. Moreover, these findings could explain the LY-OHCC’s rapid reconstitution because of the rapid water penetration through their porous matrices [30].

#### In vitro FEL release study

**Fig. 5:**
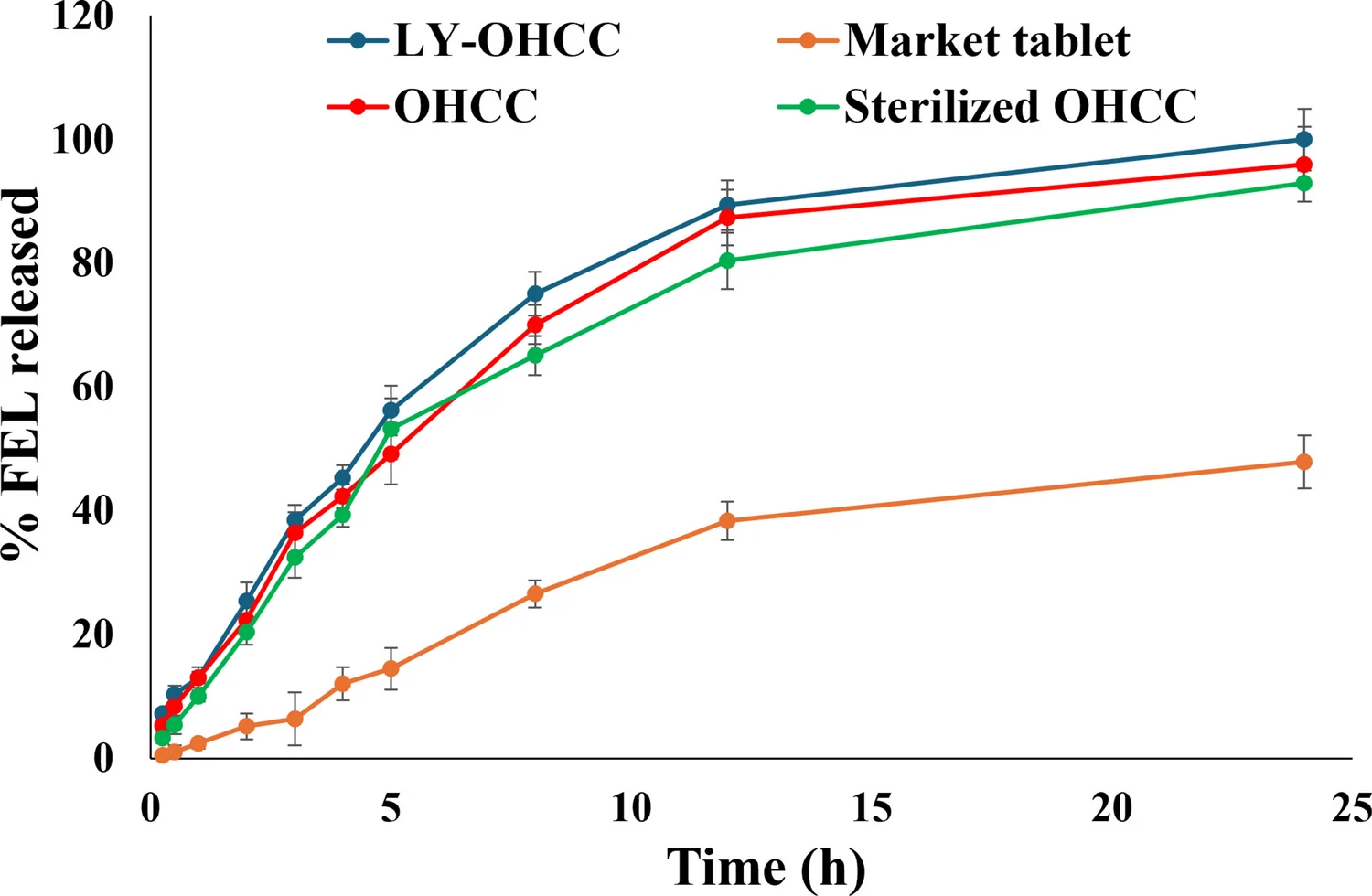
Release profiles of FEL from the OHCC formulae before, after lyophilization and after sterilization, in comparison with the market product

FEL release profiles from the OHCC formula before, after lyophilization, and after sterilization, compared to the market tablet, are illustrated in Fig. 5. After 24 h, FEL release from the LY-OHCC formula and the sterilized LY-OHCC formula were similar to the original OHCC formula with *f2* values of 70 and 69 (*p* > 0.001). This could reflect the capability of the implemented lyophilization and sterilization techniques in preserving the characteristics of the original OHCC formula [30]. This could be attributed to the high porosity of the lyophilized and sterilized formulations, absorbing a high capacity of water, resulting in fast disintegration and rapid release of the loaded nanovesicles [11, 75].

On the other hand, after 24 h, there was a significant enhancement in FEL release from the original OHCC formula before lyophilization (96.24%), LY-OHCC formula (100.05%), and sterilized LY-OHCC formula (93.51%) compared to the market tablet (47.90%), with similarity (*f2*) values of 23, 21, and 24, respectively. This reflects enhancing FEL release from the original OHCC formula before lyophilization, LY-OHCC, and sterilized LY-OHCC formula with 2.01-, 2.09-, and 1.95-fold compared to the market tablet. Moreover, there were significant differences between the drug release half-lives of the OHCC formula before lyophilization, LY-OHCC formula, and sterilized OHCC formula (6.97, 6.74, and 7.42 h, respectively) compared to the market tablet (15.07 h) (*p* < 0.001).

This enhanced release of FEL from the OHCC before and after lyophilization and sterilization could be attributed to the presence of Brij 97 that boosted FEL solubility, hence enhanced its diffusion and release into the medium. This could also be attributed to the nanosized of the loaded nanovesicles, offering a high surface area of the nanovesicles, hence high drug contact sites with the dissolving liquid, augmenting EFL solubility and facilitating its release to the medium [5, 76]. These findings agreed with Ibrahim et al., who formed FEL-loaded polymeric nanocapsules and stated that they exhibited significantly higher drug release compared with pure FEL powder. They attributed this to the nano size of the prepared polymeric nanocapsules and the lyophilization process that boosted the drug release [6]. The low FEL release from the oral market tablet could be because of the bad solubility of free FEL [1].

This is also in harmony with Sahu and Das et al., who formed lyophilized PVA based nanoparticles loaded with FEL and declared that it exhibited significantly higher FEL release compared with pure FEL powder [77].

Regarding the kinetic analysis of FEL release data from the OHCC formula, the LY-OHCC formula, and the sterilized LY-OHCC formula, it was best fitted with the Korsmeyer-Peppas model, as the highest R^2^ value appeared in this model, as displayed in Supplementary Table S1. The release exponent (n) of the OHCC formula, the LY-OHCC formula, and the sterilized LY-OHCC formula were found to be 0.76, 0.69, and 0.71, respectively, indicating a non-Fickian (anomalous) diffusion mechanism [26]. This could reveal that FEL was released due to the simultaneous diffusion of the dissolution medium into the nanovesicles and the erosion of the nanovesicular walls [78]. This behavior is in agreement with Elsayed et al.’s previous studies who developed cetyl alcohol-based nanovesicles loaded with rosuvastatin calcium and found that it followed the Korsmeyer-Peppas model with a non-Fickian (anomalous) release mechanism [39].

On the other hand, the kinetic analysis of FEL release data from the oral market tablet revealed that it was best fitted with the Higuchi diffusion model, as it had the highest R^2^ value, as displayed in Supplementary Table S1. These results indicate that FEL release from the market tablet was concentration-dependent, leading to a slow and sustained release profile [79].

#### Stability study

The sterilized LY-OHCC formula demonstrated physical stability for at least 6 months storage at room temperature. There was no significant difference between the DC, VS, PDI, ZP, and EE% values of the sterilized LY-OHCC before and after storage for 6 months, as displayed in Table 4 (*P* > 0.05). These results agree with Nayak et al. who prepared lyophilized lipid nanoparticles loaded with Curcuminoids. They reported that after storing the lyophilized optimized formula for 6 months, it maintained all the measured parameters’ values as before storage with no significant differences. They attributed this to the lyophilization technique that prevented the aggregation of nanoparticles and improved their stability [80]. This also might be due to incorporating the OHCC formula on the hydrophilic PEG chains of Brij SAA, which provided a steric barrier that prevented the nanovesicles' aggregation, stabilizing the LY-OHCC [65].

**Table 4:** The effect of storage on the sterilized LY-OHCC formula

Parameter	Fresh sterilized LY-OHCC formula	Stored sterilized LY-OHCC formula after 6 months
DC%	96.98 ± 3.17	95.80 ± 2.14
EE%	70.15 ± 4.66	69.73 ± 2.95
VS (nm)	157.08 ± 5.12	170.15 ± 4.28
PDI	0.184 ± 0.03	0.206 ± 0.03
ZP (mV)	−31.92 ± 3.21	−31.17 ± 2.77

Data are presented as mean ± SD, n = 3.

### In vitro studies using the human breast adenocarcinoma cell line (MCF-7)

#### Cell viability assay

**Fig. 6:**
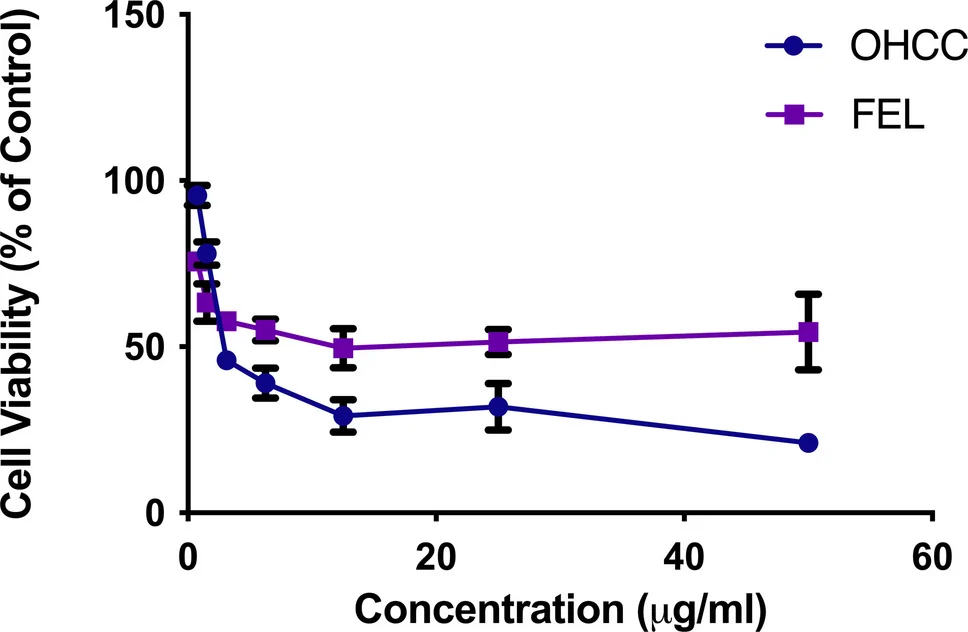
The antiproliferative effect of pure FEL and the OHCC formula against MCF-7 cells after 48 h incubation. Data are expressed as Mean S.D. (*n* = 3). The assay was carried out independently in triplicates

Ca-channel blockers exert a cytotoxic effect against different cancer cells by activating apoptosis signaling [81]. These drugs are reported to enhance the anticancer activity of different chemotherapeutic drugs such as DOX [82, 83]. We evaluated the cytotoxic effect of pure FEL and the OHCC against MCF-7 cells using the MTT assay. Both FEL and the OHCC formula significantly reduced cell viability in a concentration-dependent manner, as shown in Fig. 6. IC_50_ values were estimated for pure FEL, the OHCC, and DOX (due to the markedly lower concentration range required for doxorubicin, its dose–response curve is presented separately in Supplementary Figure SF3). The IC_50_ value of the OHCC formula (5.05 ± 1.1 µg/ml) was close to the IC_50_ value of DOX (1.66 ± 0.34 µg/ml), revealing the OHCC’s powerful anticancer activity. Moreover, the IC_50_ value of the OHCC formula showed an enhanced cytotoxic effect and suppressed the proliferation of MCF-7 cells significantly when compared to the standard form, pure FEL (33.4 ± 7.8 µg/ml). Hence, the formulation significantly enhanced the cytotoxic effect of FEL by 6.61-fold compared to pure FEL in MCF-7 cell lines.

The pronounced cytotoxicity of the OHCC formulation can be explained by the incorporation of the formula on HA, which is an active tumor-targeting ligand for the CD44 receptor, which is overexpressed in various kinds of cancers, including breast cancer. Accordingly, HA seeks tumor tissue actively and delivers FEL to the particular region efficiently [16, 17]. These results agree with Sallam et al., who prepared hyaluronic-coated cubosomes for anticancer drug delivery. They stated that HA dramatically enhanced the anticancer effect of the uncoated cubosomes in pancreatic cancer cells [18]. Ma et al. prepared PEGylated hyaluronic acid nano-drug for anticancer drug delivery. They stated that HA succeeded in achieving tumor targetability and showed a strong anticancer effect in colon cancer cells [19]. Additionally, CA is metabolized by alcohol dehydrogenase and aldehyde dehydrogenase, which are overexpressed in cancer cells [20, 21], therefore, the incorporation of CA in the formula can improve the intracellular accumulation of the drug and thus enhance its cytotoxicity.

#### Trypan blue assay for evaluating cancer cell growth inhibition

**Fig. 7:**
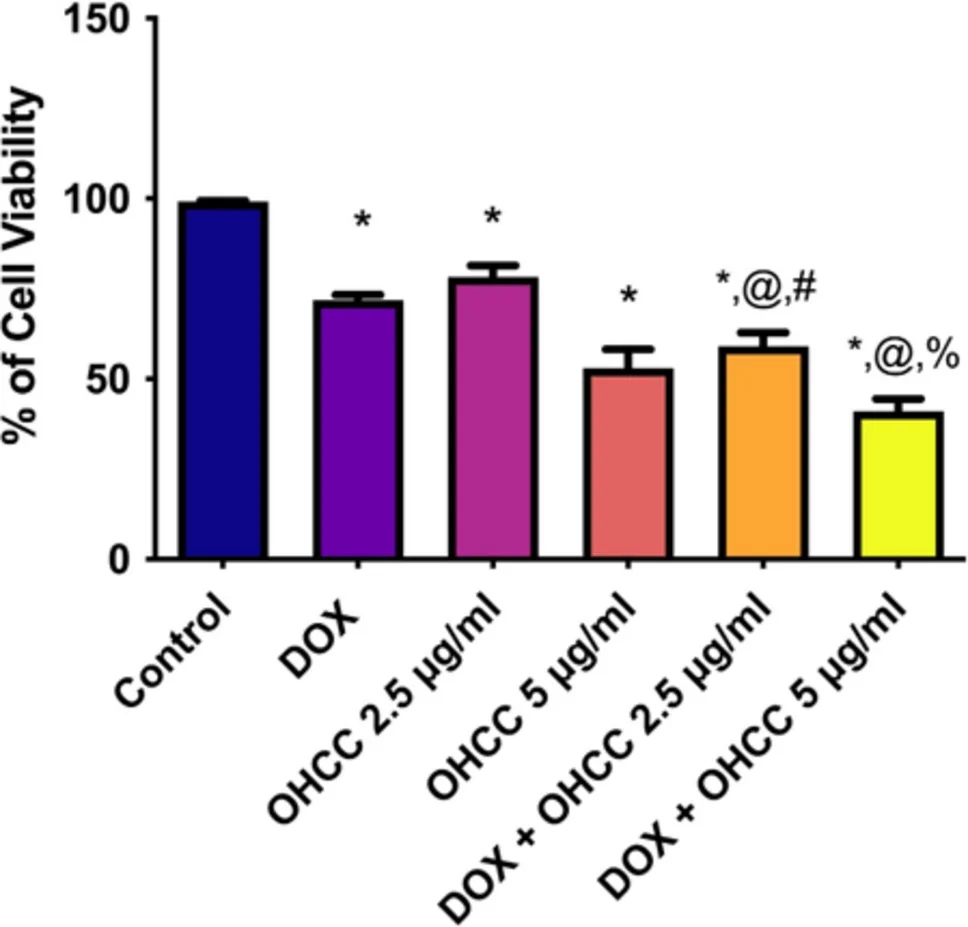
The OHCCs synergize the antiproliferative effect of DOX against MCF-7 cells. Data are expressed as Mean S.D. (*n* = 3). The assay was carried out independently in triplicate. Statistical significance was performed using one-way ANOVA. *p < 0.05 vs Control, @*p* < 0.05 vs DOX, # *p* < 0.05 vs OHCC 2.5 µg/ml, % *p* < 0.05 vs OHCC 5 µg/ml

Recent research has demonstrated that calcium channel blockers can help overcome the resistance that frequently arises to conventional anticancer medications by inducing their antiproliferative effects [81, 84]. In this study, treating MCF-7 cells with a combination of the OHCC and DOX significantly enhanced antiproliferative activity in a dose-dependent manner compared to DOX alone, as shown in Fig. 7 (*p* < 0.05). This indicates that OHCC augmented the anticancer effect of DOX against human breast cancer cells, suggesting that the active targeting of FEL sensitizes the MCF-7 cells to DOX. These findings align with previous research showing that calcium channel blockers, such as lercanidipine and amlodipine, can alleviate DOX resistance in gastric cancer cells, primarily by inhibiting oncogenic signaling and enhancing apoptotic pathways [83].

#### Fluorescent stain for evaluating the mechanism of cell death

**Fig. 8:**
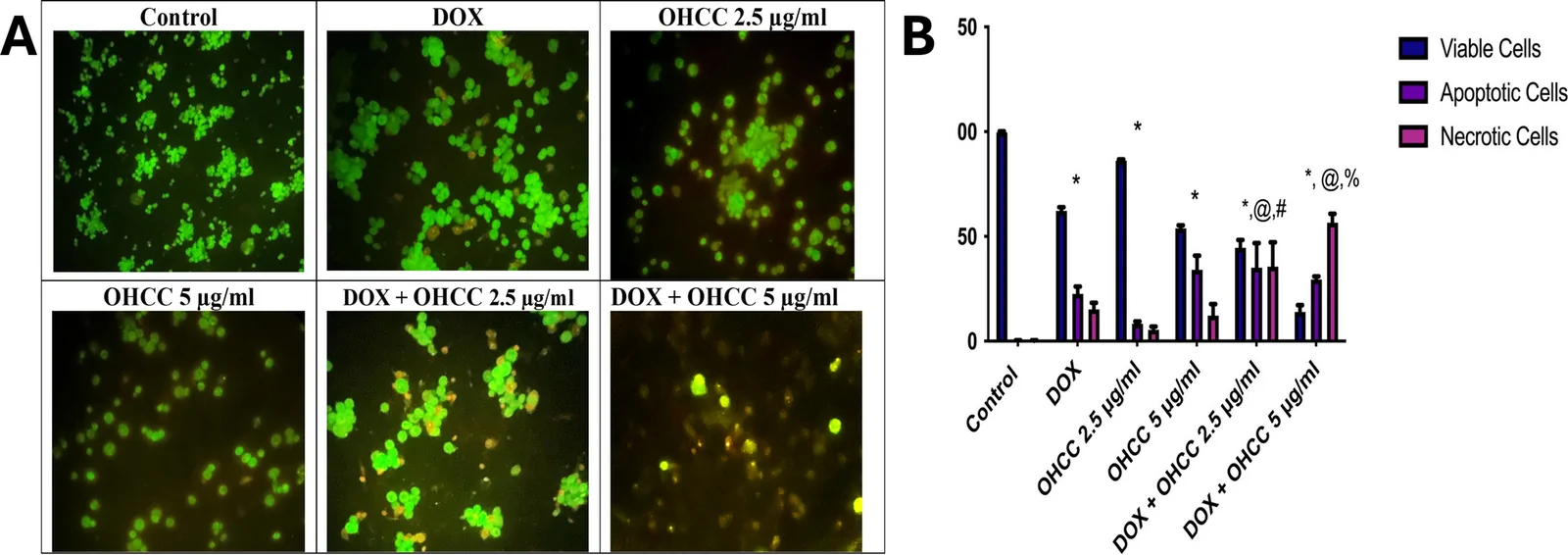
**A**) AO/EB fluorescent stain for evaluating death pattern in MCF-7 cells after incubation with DOX and/or the OHCC formula in different concentrations: live (green), apoptotic cells (yellow and light orange), and necrotic cells (dark orange and red) staining imaged by a fluorescent microscope. **B**) Cell population percentage of viable, apoptotic, and necrotic cells in MCF-7 cells of different groups. Data are expressed as mean ± standard deviation for triplicate within an individual experiment. Statistical significance was performed using one-way ANOVA. *p < 0.05 vs Control, @*p* < 0.05 vs DOX, # *p* < 0.05 vs DOX + OHCC 2.5 µg/ml, % *p* < 0.05 vs OHCC 5 µg/ml

The morphological change and the pattern of cell death induced by DOX and OHCC treatment in MCF-7 cells were evaluated by AO/EB stain. Activating apoptotic signaling is a critical target in cancer therapy, as it can effectively restrain tumor proliferation and progression. In this context, blocking L-type calcium channels (LTCCs) limits calcium influx—a process essential for regulating cell growth and apoptosis—as previously demonstrated with amlodipine in non-small lung cancer cells [85]. In the current study, the OHCC + DOX combination exhibited a significant, dose-dependent increase in both apoptotic and necrotic populations compared to DOX alone (*p* < 0.05; Fig. 8 A-B). The induced proportion of apoptotic cells suggests that the formulation promotes apoptotic pathways, potentially through enhanced intracellular DOX accumulation. These results agree with Abo Mansour et al., who declared that verapamil enhanced DOX-induced apoptosis in cancer cells by modulating mitochondrial membrane potential, inhibiting drug efflux transporters, and increasing reactive oxygen species (ROS) generation [86].

#### Bax, Bcl-2, caspase 9, and VEGF evaluation by ELISA assay

**Fig. 9:**
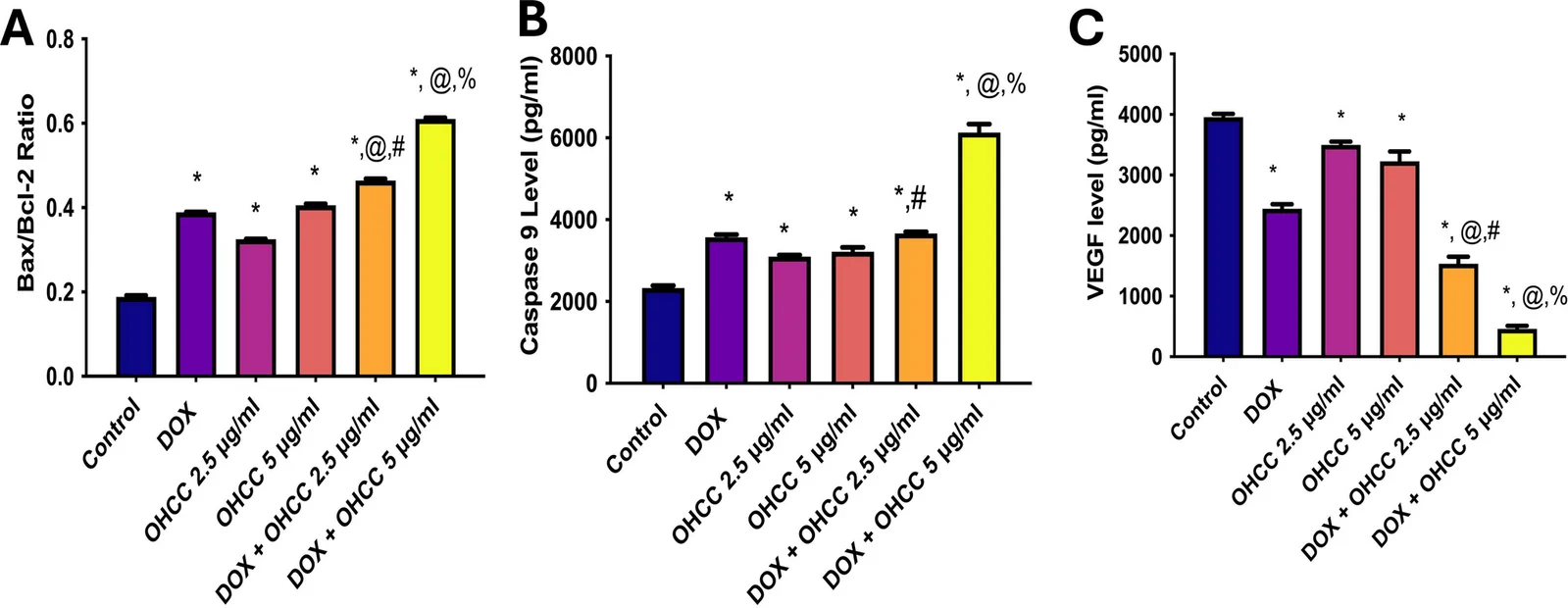
The effect of DOX and the OHCC on** A**) Bax/Bcl-2 ratio,** B**) caspase 9 level, and** C**) VEGF levels in MCF-7 cells. Data are expressed as mean ± standard deviation for triplicates within an individual experiment. Statistical significance was performed using one-way ANOVA. *p < 0.05 vs Control, @*p* < 0.05 vs DOX, # *p* < 0.05 vs DOX + OHCC 2.5 µg/ml% *p* < 0.05 vs OHCC 5 µg/ml

The ELISA results demonstrated that OHCC synergized with DOX in a dose-dependent manner, increasing the Bax/Bcl-2 ratio compared to DOX alone (*p* < 0.05; Fig.  9 A), This shift toward a higher proapoptotic-to-anti-apoptotic protein ratio indicates enhanced activation of the mitochondrial apoptotic pathway.

Similarly, caspase-9 levels were markedly elevated in the OHCC + DOX combination-treated MCF-7 cells when compared to DOX alone (*p* < 0.05; Fig. 9B). This further confirms activation of the intrinsic apoptosis cascade, as caspase-9 is a key initiator caspase in the mitochondrial pathway. These results agree with Alqudah et al., who stated that amlodipine induces caspase-dependent apoptosis in breast cancer cells [87].

Angiogenesis in breast cancer is a key factor for growth and metastasis; therefore, antiangiogenic therapy is a highly promising treatment avenue [88]. In the current study, VEGF expression was significantly suppressed in MCF-7 cells treated with OHCC + DOX compared to control and DOX-alone groups (*p* < 0.05; Fig.  9 C). This suppression in VEGF is consistent with emerging evidence that calcium channel blockers can modulate angiogenic signaling. Nifedipine has been shown to inhibit the transient receptor potential canonical (TRPC1) ion channel and the inflammatory markers, thereby regulating VEGF expression in ovarian models [89]. The reduction in VEGF levels suggests that the OHCC–DOX combination not only promotes the apoptotic pathway but also exerts a strong antiangiogenic effect. This dual action highlights the potential of the OHCC–DOX combination to limit both tumor growth and vascular support.

### In vivo studies

#### Tumor volume, growth inhibition rate, and weight

**Fig. 10:**
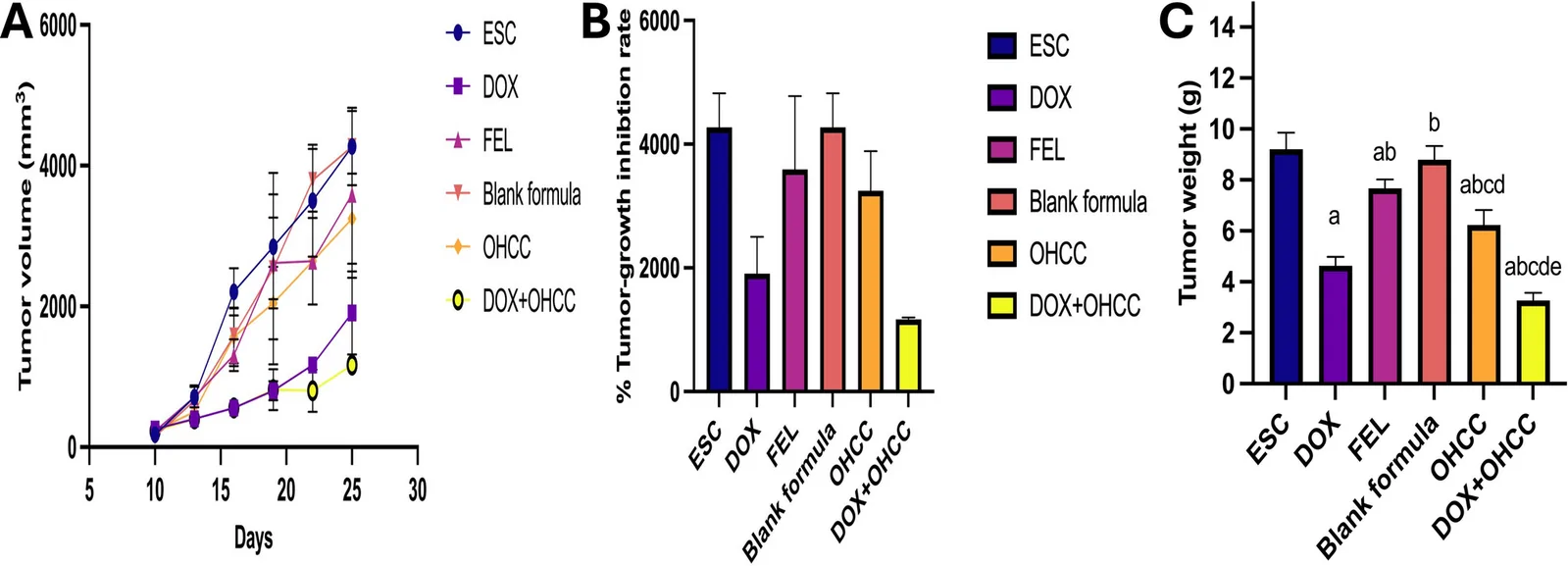
Effect of DOX, FEL, blank formula, FEL-OHCC, and DOX + FEL-OHCC on** A**) Tumor volume,** B**) % Tumor-growth inhibition, and **C**) Tumor weight in ESC-bearing mice. One-way ANOVA was used, and Tukey’s multiple comparisons test followed for significance. ^a^ significantly different from ESC control, ^b^ significantly different from DOX, ^c^ significantly different from FEL, ^d^ significantly different from blank formula, and.^e^ significantly different from FEL-OHCC (*p* < 0.05)

As shown in Fig.  10 A, treatment of ESC-bearing mice with DOX, FEL, OHCC, or the DOX + OHCC combination significantly inhibited tumor growth compared to the untreated ESC control group. The most potent effect was observed with the DOX + OHCC combination, which achieved a 73% inhibition of tumor growth rate, significantly greater than DOX alone (55%) (Fig. 10B). Measurement of tumor weights at the end of the experiment further confirmed these findings (Fig.  10 C). All treated groups displayed a significant reduction in tumor weight compared to the control ESC group, with the greatest reduction recorded in the DOX + OHCC group (65%), followed by DOX alone (50%). FEL alone produced a modest effect, reducing tumor weight by 17%, whereas OHCC treatment resulted in a more substantial 33% reduction, both statistically significant relative to the ESC group.

The enhanced antitumor efficacy observed with OHCC may also be attributed to the calcium channel blockade's ability to modulate the tumor microenvironment. Calcium signaling plays a pivotal role in tumor angiogenesis, stromal activation, and cancer–immune cell interactions. Inhibition of calcium influx has been shown to suppress vascular endothelial growth factor (VEGF) expression and impair neovascularization, thereby limiting tumor growth and metastatic potential [90, 91]. Furthermore, CCBs have been reported to reduce cancer-associated fibroblast activation and extracellular matrix remodeling, which may further contribute to tumor growth suppression in vivo [92]. These mechanisms provide a plausible explanation for the superior tumor inhibition observed with OHCC compared to free FEL.

These results align with previous evidence supporting the anticancer potential of calcium channel blockers (CCBs). FEL, as an L-type calcium channel blocker, is associated with intracellular calcium buildup, which promotes apoptosis and sensitizes cancer cells to chemotherapy [93–96]. Jacquemet et al. (2016) further reported that the L-type calcium channels regulate cancer cell invasion via downstream signaling of integrin and identified FEL as one of the potent inhibitors of filopodia formation in cancer cells [97]. More recently, Liang and Xiao (2023) highlighted the anticancer effects of pure FEL after reporting the impairment of the proliferative and migratory abilities of cancer cells post-treatment [98]. In agreement with these findings, our study is the first to report the cytotoxic effects of the FEL hyaluronic acid-coated cetylosomal formula, OHCC, which showed superior anticancer activity compared to unformulated FEL.

Moreover, the enhanced efficacy of OHCC and its synergistic effect with DOX can be attributed to several key physicochemical properties. The nanoscale vesicle size (157.08 nm) favors tumor penetration and accumulation via the enhanced permeability and retention (EPR) effect, consistent with reports that nanoparticles below 200 nm efficiently accumulate within tumor tissue [99, 100]. The high negative zeta potential (−31.92 mV) of the OHCC further contributed to its tumor penetration and retention, in line with studies demonstrating that nanoparticles with charges greater than + 10 mV or below − 30 mV show superior cellular uptake and reduced efflux [101–103]. In addition, incorporation of Brij 97 may have enhanced FEL permeation through cancer cell membranes [104]. Finally, the dual incorporation of HA and CA in the formulation provided active targeting, as HA binds to CD44 receptors overexpressed in tumor cells, while CA improves stability and membrane interaction [16, 17, 20, 21]. Together, these properties support the selective, rapid, and efficient uptake of OHCC by cancer cells, which in turn potentiates the cytotoxic activity of DOX in vivo.

Importantly, the observed synergy between DOX and OHCC may also be explained by FEL-mediated chemosensitization. Calcium channel blockade has been shown to inhibit P-glycoprotein (P-gp) and other ATP-binding cassette (ABC) transporters, which are key contributors to multidrug resistance in cancer cells [105–107]. By impairing drug efflux mechanisms, FEL may enhance intracellular retention of DOX, thereby amplifying its cytotoxic effect. Additionally, FEL-induced disruption of calcium homeostasis can potentiate DOX-triggered mitochondrial dysfunction and reactive oxygen species (ROS) generation, leading to enhanced apoptosis [108, 109]. This multifactorial interaction likely underlies the superior tumor growth inhibition observed with the DOX + OHCC combination.

##### Survival time and percent increase in life span

The antiproliferative efficacy of DOX, FEL, blank formula, OHCC, and DOX + OHCC in ESC-bearing mice was further evaluated by mean survival time (MST) and percentage increase in life span (%ILS). As shown in Table 5, the ESC control group had a mean survival of 19 days. Treatment with DOX extended MST to 24 days (%ILS = 26.3%), while pure FEL alone produced a modest increase to 20.5 days (%ILS = 7.9%). In contrast, mice treated with OHCC survived an average of 22 days (%ILS = 15.8%), demonstrating improved efficacy over pure FEL. Notably, the DOX + OHCC combination achieved the greatest effect, with an MST of 24.5 days and a %ILS of 28.9%, which was significantly higher than either DOX or OHCC alone. As displayed in Table 5, treatment with DOX or DOX + OHCC resulted in a T/C% greater than 125%, confirming their meaningful extension of survival.

**Table 5:** Mean survival time (MST), percentage increase in life span (%ILS), and mean survival time of treated mice/mean survival time of control mice (T/C%) of different treatment groups in Ehrlich-bearing mice

Group	MST	%ILS	T/C%
ESC	19	––––	––––
DOX	24	26.3	126.3
FEL	20.5	7.9	108
Blank formula	19.5	2.6	102.6
OHCC	22	15.8	115.8
DOX + OHCC	24.5	28.9	130

These findings align with clinical and preclinical reports linking CCBs to improved cancer outcomes. For example, a retrospective study showed that CCBs, particularly when combined with aspirin, improved survival in patients with resected pancreatic ductal adenocarcinoma [110]. Later, Tingle et al. (2020) also reported that CCB monotherapy was associated with prolonged overall survival in unresectable pancreatic cancer patients [111]. More recently, Liang and Xiao (2023) highlighted that FEL treatment correlates positively with overall and disease-free survival, suggesting an intrinsic anticancer benefit [98]. In the present study, FEL alone conferred only a modest improvement in survival, but its formulation into OHCC substantially enhanced efficacy. Furthermore, combining OHCC with DOX produced the most pronounced survival benefit, emphasizing the synergistic advantage of the formulation.

The marked improvement in survival observed with OHCC and, more prominently, with the DOX + OHCC combination underscores the clinical relevance of nanoparticle-based drug repurposing strategies. Nanocarrier systems are known to improve pharmacokinetics, prolong systemic circulation, and reduce off-target toxicity, which collectively contribute to improved therapeutic outcomes and survival [112, 113]. In the context of DOX therapy, nanoparticle-mediated delivery has been shown to mitigate cardiotoxicity while maintaining or enhancing antitumor efficacy, thereby allowing sustained treatment and improved survival [114, 115]. Although cardiotoxicity was not directly assessed in the present study, the survival advantage observed with the DOX + OHCC group may partially reflect improved tolerability and reduced systemic burden.

Collectively, these findings support the concept that FEL-loaded nanocarriers not only enhance intrinsic anticancer activity but also function as effective chemosensitizers when combined with conventional chemotherapeutic agents such as DOX. The significant extension of survival achieved with the DOX + OHCC combination highlights its potential as a promising therapeutic strategy, warranting further investigation and possible translational development.

#### Histopathological findings

**Fig. 11:**
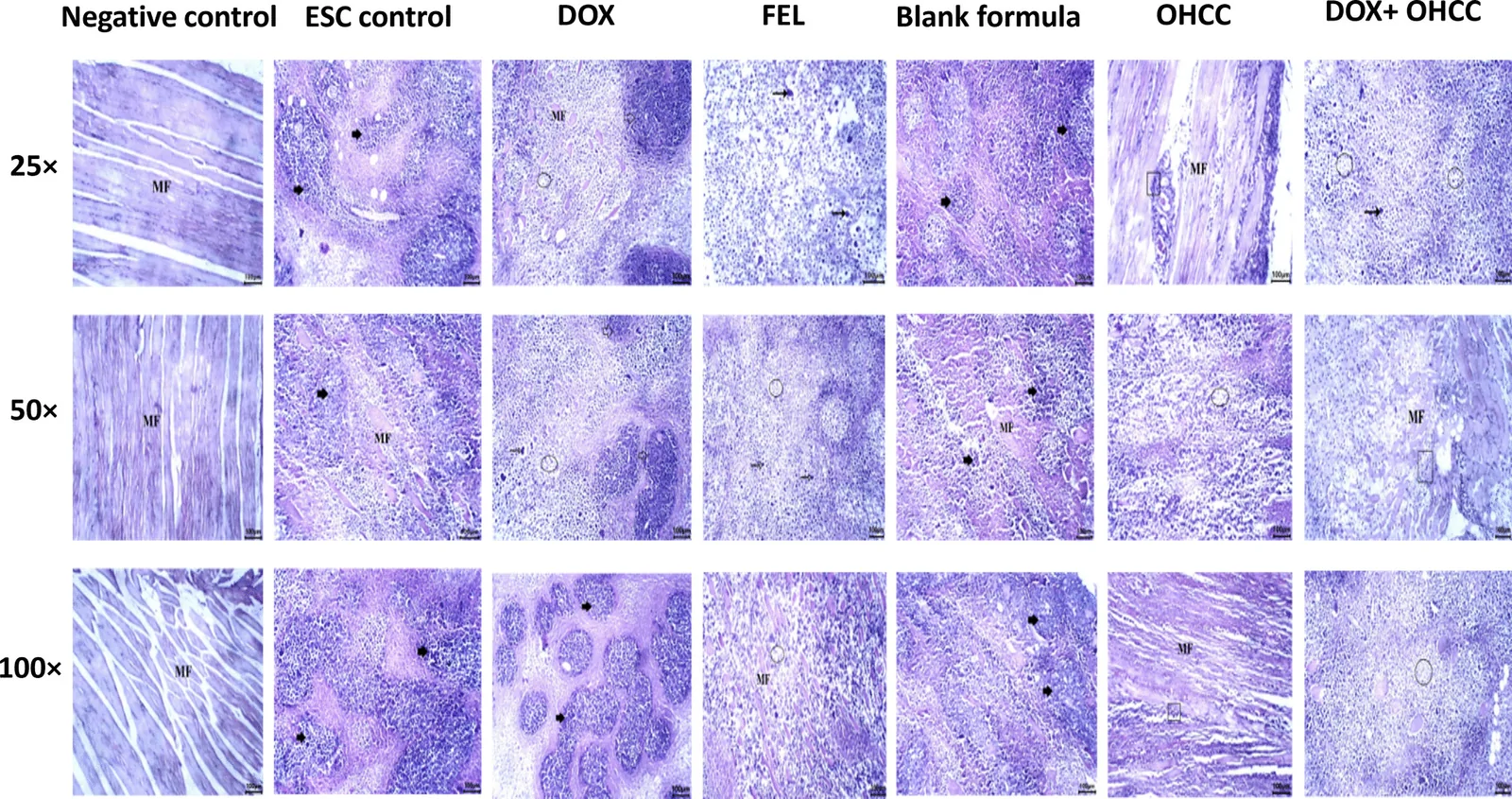
Histopathological photomicrographs of Untreated control, ESC control, DOX, FEL, Blank formula, OHCC, and DOX + OHCC groups. H&E stain (original magnifications 25x, 50 × and 100x)

Histological analysis revealed distinct differences among treatment groups (Fig. 11). In the healthy negative control group, the skeletal muscles displayed typical muscular fibers (MF) characterized by well-defined borders and peripherally positioned nuclei. In contrast, the ESC control group exhibited an infiltrating tumor consisting of sheets and nodules containing well-differentiated viable tumor cells (bold arrow). These cells displayed hyperchromatic nuclei that infiltrated between the damaged MF. The tumor typically manifests as a solid mass exhibiting an uneven or lobulated morphology. Expansive growth may occur, resulting in the invasion of adjacent muscle tissues. The structural arrangement of muscle fibers is aberrant, characterized by uneven and disorganized fibers that may exhibit a dispersed or infiltrated appearance due to the presence of tumor cells.

In DOX-treated mice, skeletal muscles exhibited significant structural damage, including swelling, uneven and fuzzy cell boundaries, bright eosinophilic cytoplasm, and a small region of necrotic tumor tissue (oval shape). A concentrated mass of cells from the solid tumor tissue (bold arrow), which displayed considerable dissemination and penetration into the muscle layer.

The FEL group demonstrated modest tumor reduction, with large necrotic zones (oval shape), apoptotic bodies, and multinucleated giant cells (thin arrow), indicating DNA damage and aberrant mitotic activity.

The photomicrographs of the blank formula group were similar to those of the ESC control group.

The OHCC group showed more pronounced effects, with marked tumor regression, extensive apoptotic regions (oval shape), and residual necrotic cells. Tumor infiltration appeared less cohesive, and evidence of karyopyknosis and karyolysis confirmed apoptosis. Interestingly, infiltration of inflammatory cells (square), such as lymphocytes or macrophages, suggested an active immune response to tumor clearance.

The combination group (DOX + OHCC) exhibited the most significant tumor regression, characterized by widespread apoptosis, necrosis (oval shape), and a reduced density of viable tumor cells. Large multinucleated cells were observed, consistent with treatment-induced DNA damage leading to defective mitosis. The presence of inflammatory infiltrates further supports the notion that the therapy induced both direct cytotoxicity and host-mediated antitumor responses.

Ultimately, these histological and morphological changes correspond to increased levels of pro-apoptotic markers and reduced VEGF, underscoring the significance of OHCC in enhancing both cytotoxic and antiangiogenic mechanisms in vivo.

## Conclusion

FEL was repurposed in this study as an anticancer drug due to the inclusion of 1,4-DHPs. FEL-HCCs were prepared using a thin-film hydration technique according to the Box-Behnken design. The OHCC formula was composed of 100 mg Brij 97, 32.44 mg cetyl alcohol (CA), and 10 mg hyaluronic acid (HA). This OHCC formula showed the highest desirability value (0.626). It had the maximum EE% and ZP, and the minimum VS and PDI. The TEM micrographs revealed spherical-shaped vesicles with a smooth surface and uniform distribution. The SEM images showed the high porosity of the examined matrices. The lyophilization and sterilization techniques did not impact the OHCC formula’s characteristics. The OHCC formula enhanced FEL release by 2.01-fold compared to the FEL market tablet, with no significant difference between the OHCC’s release before and after lyophilization and sterilization. The sterilized OHCC was stable for up to 6 months at room temperature. Moreover, the OHCC formulation exhibited significantly improved cytotoxicity against MCF-7 breast cancer cells by 6.61-fold compared to free FEL. It also enhanced the anticancer effect of DOX by modulating key apoptotic markers and suppressing VEGF expression. In vivo studies confirmed the therapeutic efficacy of the OHCC formulation, showing marked inhibition of tumor growth with a concurrent increase in both apoptotic and necrotic cancer cell death, particularly when used in combination with DOX. Moreover, the histopathological investigation supported the apoptotic and necrotic death of cancer cells. Subsequently, it could be considered that the OHCC formulation offers a promising nanocarrier system for the targeted parenteral delivery of FEL, demonstrating significant potential for cancer therapy through enhanced drug solubility and synergistic antitumor efficacy via chemosensitizing therapy. From a translational perspective, the favorable physicochemical properties, storage stability, and enhanced in vivo performance of the OHCC formulation underscore its suitability for further preclinical development. Future studies should focus on detailed pharmacokinetic profiling, long-term safety evaluation, and assessment of cardioprotective potential when combined with anthracyclines to fully elucidate its clinical applicability. Collectively, these findings position OHCC as a versatile nanoplatform for drug repurposing strategies and combination cancer therapy.

Supplementary Figure SF1: show the effect of significant interactions of SAA Amount (X_1_) and CA amount (X_2_) on Vesicle Size.

Supplementary Figure SF2: show the effect of significant interactions of SAA Amount (X_1_) and CA amount (X_2_) on Polydispersity index.

Supplementary Figure SF3: Dose–response curve of doxorubicin (DOX) in MCF-7 breast cancer cells following 48 h treatment, assessed by the MTT assay. DOX was tested over a concentration range of 20–0.156 µg/mL due to its high cytotoxic potency. Data are presented as mean ± SD (*n* = 3). The IC₅₀ value was calculated using nonlinear regression analysis.

## Supplementary Information

Below is the link to the electronic supplementary material.

ESM 1 (TIF 675 KB)

ESM 2 (TIF 143 KB)

ESM 3 (TIF 320 KB )

ESM 4 (DOCX 21.9 KB)

## Data Availability

All the datasets of the current study are available from the corresponding author upon a reasonable request.
