# Supplementary material for: Repurposing felodipine via hyaluronic acid-coated cetylosomes (HCCs) for parenteral active targeting of cancer: Box-Behnken statistical optimization, in vitro characterization, and in vivo studies
Source: Drug Deliv Transl Res. 2026 Apr 6;16(11):4155–77. doi: 10.1007/s13346-026-02090-0 (PMC13748057; doi:10.1007/s13346-026-02090-0)
Supplement: Supplementary file 1 [file 13346_2026_2090_MOESM4_ESM.docx]

***Supplementary Table (ST1): Kinetic Analysis of the Release Data of FEL from the OHCC formula, the LY-OHCC formula, the sterilized LY-OHCC formula, and the oral market tablet.***

| Systems Code | Model | R^2^ | Mechanism of Release |
| --- | --- | --- | --- |
| OHCC | Zero Order | 0.8085 | **Korsemeyer–Peppas model** |
|  | First Order | 0.6076 |  |
|  | Higuchi diffusion | 0.9412 |  |
|  | Korsemeyer–Peppas | 0.9735 |  |
| LY-OHCC | Zero Order | 0.7977 | **Korsemeyer–Peppas model** |
|  | First Order | 0.5956 |  |
|  | Higuchi diffusion | 0.9363 |  |
|  | Korsemeyer–Peppas | 0.9623 |  |
| Sterilized LY-OHCC | Zero Order | 0.8085 | **Korsemeyer–Peppas model** |
|  | First Order | 05631 |  |
|  | Higuchi diffusion | 0.9445 |  |
|  | Korsemeyer–Peppas | 0.9606 |  |
| Oral market tablet | Zero Order | 0.9134 | **Higuchi diffusion model** |
|  | First Order | 0.6842 |  |
|  | Higuchi diffusion | 0.9605 |  |
|  | Korsemeyer–Peppas | 0.9534 |  |

*OHCC is the optimized hyaluronic acid-coated cetylosomal formula. LY-OHCC means the lyophilized optimized hyaluronic acid-coated cetylosomal formula. Data presented as mean average value ± SD.
